# Short-Term Silymarin Supplementation After Kidney Transplantation Is Associated with Reduced Early Albuminuria: A Prospective Cohort Study

**DOI:** 10.3390/jcm15176694

**Published:** 2026-08-28

**Authors:** Matej Vnučák, Karol Graňák, Patrícia Kleinová, Tímea Jusková, Andrej Kollár, Katarína Ševčíková, Ivana Dedinská

**Affiliations:** 1 Transplant-Nephrology Department, University Hospital Martin, Kollárova 2, 036 59 Martin, Slovakia; 2 I. Department of Internal Medicine, Jessenius Faculty of Medicine in Martin, Comenius University in Bratislava, Malá Hora 10701/41, 036 01 Martin, Slovakia

**Keywords:** kidney transplantation, silymarin, albuminuria, early post-transplant outcomes

## Abstract

**Background:** Early post-transplant albuminuria is associated with adverse graft and cardiovascular outcomes and may reflect ischemia–reperfusion injury-induced damage to the glomerular filtration barrier. However, evidence for safe adjunctive interventions targeting these early pathophysiological processes remains limited. **Methods:** In this single-center prospective cohort study with historical controls, adult kidney transplant recipients transplanted between January 2020 and September 2022 served as controls, while consecutive recipients transplanted from October 2022 onward received adjunctive silymarin (900 mg/day for 30 days) in addition to standard immunosuppression. The principal renal outcome was UACR assessed during the first 3 months post-transplant. A ≥25% reduction in UACR between Months 1 and 3 was additionally evaluated as an exploratory responder outcome. Secondary outcomes included estimated glomerular filtration rate (eGFR), lipid profile parameters, tacrolimus exposure, and safety. **Results:** A total of 136 patients (78 silymarin-treated and 58 controls) were included. UACR was consistently lower in the silymarin group at months 1–3. In multivariable analysis, silymarin treatment was independently associated with lower UACR at month 3. In an exploratory responder analysis, a ≥25% reduction in UACR between Months 1 and 3 occurred more frequently in silymarin-treated patients (OR 3.28, 95% CI 1.38–7.79; *p* = 0.007). eGFR trajectories were similar between groups. No consistent independent effects on lipid parameters were observed after adjustment. Tacrolimus exposure and adverse event rates were comparable between groups. **Conclusions:** Short-term silymarin exposure initiated early after kidney transplantation was associated with lower UACR during the first three post-transplant months. These findings support a potential targeted effect on early glomerular injury and suggest that silymarin may represent a safe adjunctive strategy to modulate early post-transplant risk.

## 1. Introduction

Early post-transplant alterations in albuminuria and lipid metabolism are common in kidney transplant recipients and have been consistently associated with adverse long-term graft and cardiovascular outcomes. Despite advances in immunosuppressive therapy, cardiovascular disease remains a leading cause of mortality in this population, highlighting the need for strategies targeting non-immunologic risk pathways.

Albuminuria reflects early glomerular and endothelial injury, while dyslipidemia contributes to increased cardiovascular risk and chronic allograft dysfunction. However, safe and readily applicable interventions targeting these early cardiometabolic risk markers in the immediate post-transplant period remain limited.

Ischemia–reperfusion injury (IRI), an inevitable consequence of kidney transplantation (KT), represents one of the principal mechanisms underlying early allograft injury. Excessive generation of reactive oxygen species during reperfusion induces lipid peroxidation, endothelial dysfunction, inflammatory activation, and apoptosis of renal cells, resulting in damage to both tubular epithelial cells and the glomerular filtration barrier [1]. Malondialdehyde (MDA), a marker of lipid peroxidation, and tumor necrosis factor-α (TNF-α), a key pro-inflammatory cytokine, are significantly increased during the early post-transplant period and are considered major mediators of IRI-related tissue injury [2,3]. Experimental evidence further suggests that IRI directly contributes to disruption of podocyte integrity, endothelial glycocalyx injury, and microvascular dysfunction, ultimately leading to increased glomerular permeability and albuminuria [4].

Silymarin is a polyphenolic flavonoid complex composed predominantly of flavonolignans, with silibinin (silybin) as its principal bioactive component, exhibiting antioxidant and lipid-modulating properties [5]. It has been widely used for its hepatoprotective effects in alcoholic and nonalcoholic fatty liver disease and cirrhosis, with additional biological effects including membrane stabilization, free radical scavenging, and inhibition of inflammatory and fibrogenic pathways [6].

In experimental models, silymarin attenuates oxidative stress and inflammatory signaling by reducing lipid peroxidation, MDA production, and TNF-α expression while suppressing endothelial activation through downregulation of adhesion molecules such as vascular cell adhesion molecule-1 and intercellular adhesion molecule-1 [7]. In a randomized clinical trial of patients with diabetic nephropathy, Fallahzadeh et al. demonstrated significant reductions in urinary albumin excretion accompanied by decreases in circulating TNF-α and MDA following silymarin supplementation [8]. Meta-analyses further suggest that silymarin may exert nephroprotective effects in chronic kidney disease, although the available clinical evidence remains heterogeneous and inconclusive. Experimental studies have also demonstrated protective effects against calcineurin inhibitor-induced nephrotoxicity; however, these findings have not yet been confirmed in human kidney transplant recipients.

Beyond hepatic disease, meta-analyses of clinical trials suggest that silymarin may exert nephroprotective effects, although evidence in chronic kidney disease remains heterogeneous and largely inconclusive, particularly with respect to albuminuria [9]. In the transplant setting, experimental studies have demonstrated protective effects of silymarin against calcineurin inhibitor-induced nephrotoxicity; however, these findings have not been confirmed in human transplant populations [10].

In this context, we conducted a prospective cohort study with historical controls in a well-characterized population of kidney transplant recipients managed under a uniform clinical protocol, including standardized immunosuppressive therapy, follow-up schedules, and laboratory assessments. This design enabled us to capture early post-transplant dynamics in albuminuria and cardiometabolic parameters within a consistent clinical framework.

We hypothesized that attenuation of IRI-induced oxidative stress and inflammation by short-term silymarin supplementation initiated immediately after kidney transplantation would reduce injury to the glomerular filtration barrier, resulting in lower albuminuria during the early post-transplant period, without adversely affecting graft function or immunosuppressive therapy.

## 2. Material and Methods

### 2.1. Study Design and Population

The Silymarin’s Advantage on Graft Effectiveness (SAGE) study was a single-center prospective cohort study with historical controls conducted at the Transplant-Nephrology Department, University Hospital Martin, Slovakia. All consecutive kidney transplant recipients who provided written informed consent were eligible for inclusion. Early graft loss or patient death were not exclusion criteria and contributed to baseline and safety analyses. Patients lacking month-3 urine albumin-to-creatinine ratio (UACR) measurements were excluded from primary endpoint analysis.

Recipients transplanted between January 2020 and September 2022 who received standard immunosuppression constituted the control cohort, whereas consecutive recipients transplanted from October 2022 onward received adjunctive silymarin (900 mg/day for 30 days) in addition to the same immunosuppressive regimen. Because silymarin supplementation was implemented as part of the institutional post-transplant protocol from October 2022 onward, a contemporaneous untreated control group was not available; therefore, consecutive recipients transplanted immediately before protocol implementation served as historical controls. Follow-up was censored on 31 July 2025, ensuring a minimum follow-up of three months for all included patients. Throughout the study period, institutional standards of care remained unchanged, including donor selection and acceptance criteria, perioperative management, immunosuppressive protocols, biopsy indication criteria, laboratory assays and analytical methods, concomitant medication practices, and scheduled follow-up procedures. Thus, apart from the introduction of adjunctive silymarin in October 2022, no intentional changes in the early post-transplant management protocol were introduced between the two study periods.

The study was investigator-initiated and received Institutional Ethics Committee approval (EK 21/2022) before enrolment of the prospective cohort. The original study concept considered a randomized controlled design; however, practical and regulatory considerations before implementation led to adoption of the prospective cohort design with historical controls that was ultimately conducted. The study was retrospectively registered at ClinicalTrials.gov (NCT06801886) on 24 January 2025. The registry entry retained elements of the originally planned randomized design and therefore does not fully represent the final implemented methodology. The study was reported in accordance with the Transparent Reporting of Evaluations with Nonrandomized Designs (TREND) statement; the completed TREND checklist is provided in the Supplementary Materials.

Detailed methodological descriptions are provided in the Supplementary Methods S1A.

### 2.2. Donor and Recipient Characteristics

Recipient demographic, clinical, donor, and immunological characteristics were prospectively recorded according to a predefined study protocol. Recorded recipient characteristics included age, sex, body mass index (kg/m^2^), transplant history, etiology of end-stage kidney disease, dialysis modality and duration, and waiting time for KT. The major comorbidities and donor characteristics were systematically documented. Extended criteria donor (ECD) status was defined according to United Network for Organ Sharing (UNOS) criteria [11].

Immunological assessment included human leukocyte antigen (HLA) mismatches and recipient sensitization status, evaluated by panel-reactive antibody levels and pre-transplant antibody screening. Donor-specific antibodies (DSA) were identified based on donor HLA typing. Detailed definitions, laboratory methods, and immunological parameters are provided in Supplementary Methods S1A.

### 2.3. Immunosuppressive Treatment

All recipients received perioperative methylprednisolone and risk-adapted induction immunosuppression consisting of basiliximab in low-risk patients and antithymocyte globulin in moderate- to high-risk patients, according to a standardized institutional protocol.

Maintenance immunosuppression consisted of tacrolimus (TAC), mycophenolic acid (MPA), and prednisone, with TAC doses titrated to target trough levels and prednisone tapered according to institutional standard practices. A detailed description of both induction and maintenance immunosuppressive protocols, including risk stratification criteria and dosing schedules, is provided in Supplementary Methods S1A.

### 2.4. Concomitant Medication

During the study period, none of the participants received sodium–glucose cotransporter-2 inhibitors (SGLT2i) or renin–angiotensin–aldosterone system inhibitors (angiotensin-converting enzyme inhibitors or angiotensin receptor blockers). According to our institutional post-transplant protocol, these agents are generally not initiated within the first three months after kidney transplantation because of the dynamic recovery of graft function and the need for frequent adjustment of immunosuppressive therapy. Therefore, these medications could not have influenced urinary albumin excretion during the study period.

### 2.5. Investigation Algorithm

Clinical and laboratory assessments followed a predefined protocol covering the perioperative and early post-transplant periods. Routine biochemical testing, including kidney function, metabolic parameters, lipid profile, hepatic enzymes, and UACR, was performed at transplantation, on postoperative day 4, and during scheduled outpatient visits at months 1 and 2. Baseline kidney function was defined using the average creatinine values from postoperative days 4–7 to minimize early postoperative variability. Delayed graft function (DGF) was defined as the requirement for dialysis within the first 7 days after kidney transplantation. Pre-transplant UACR assessment was not performed because urine production before transplantation was highly heterogeneous. Many recipients were oliguric or anuric due to end-stage kidney disease, whereas others retained variable residual diuresis. Consequently, a standardized pre-transplant urine albumin-to-creatinine ratio could not be obtained or meaningfully compared across participants. Therefore, the first standardized assessment was scheduled one month after transplantation, when all recipients had established graft function and reliable spot urine sampling was feasible.

At 3 months, all recipients underwent protocol graft biopsy and donor-specific antibody assessment, accompanied by comprehensive laboratory evaluation. For-cause graft biopsies and antibody testing were performed at any time during follow-up in cases of graft dysfunction, new-onset or worsening proteinuria, or the detection of de novo donor-specific antibodies. Biopsy indications and histopathological assessments followed predefined criteria and remained unchanged throughout the study period.

Data on maintenance immunosuppression, lipid-lowering therapy, antidiabetic treatment, and surgical complications were collected systematically. Post-transplant diabetes mellitus was diagnosed according to the American Diabetes Association criteria, whereas surgical complications were classified according to the criteria described by Humar et al. [12,13]. Detailed definitions are provided in Supplementary Methods S1A. 

### 2.6. Primary and Secondary Outcomes

The principal renal outcome was UACR assessed during the first three months after KT, with standardized measurements obtained at Months 1 (M1), 2, (M2) and 3. Because the first standardized UACR measurement was obtained at Month 1, following the 30-day silymarin exposure period, it was not considered a pretreatment baseline measurement. UACR was analyzed as a continuous outcome across the available post-transplant assessments. The proportion of recipients achieving a ≥25% reduction in UACR between Months 1 and 3 was additionally evaluated as an exploratory responder analysis of the subsequent post-treatment UACR trajectory.

Secondary renal outcomes included graft function recovery assessed by eGFR at M3 adjusted for eGFR at M1, and the proportion of patients achieving a ≥10% increase in eGFR between M1 and M3.

Secondary metabolic outcomes included changes in lipid profile parameters, specifically low-density lipoprotein (LDL) cholesterol, HDL (high-density lipoprotein cholesterol) cholesterol, and non-HDL (non-high-density lipoprotein cholesterol) cholesterol, analyzed as M3 values adjusted for corresponding M1 values. Clinically meaningful lipid changes were additionally evaluated as binary outcomes, including ≥15% reduction in LDL cholesterol or non-HDL cholesterol and ≥15% increase in HDL cholesterol between M1 and M3. Sensitivity analyses additionally adjusted lipid models for initiation of lipid-lowering therapy during follow-up.

Additional secondary outcomes included early metabolic complications within the 3-month follow-up period, specifically de novo post-transplant diabetes mellitus (PTDM) and impaired glucose metabolism. PTDM was diagnosed according to the 2025 American Diabetes Association criteria [13]. Differences in mean TAC trough concentrations between the groups were assessed during follow-up. Immunologic outcomes included development of de novo DSA and biopsy-proven acute rejection. Safety outcomes included adverse events and tolerability of silymarin supplementation.

### 2.7. Silymarin Supplementation

Silymarin was administered exclusively to the intervention group at a total daily dose of 900 mg for 30 consecutive days after KT. This regimen consisted of six tablets per day, each containing 240 mg of dry extract from milk thistle fruit (Cardui mariae fructus extractum siccum), equivalent to 150 mg of silymarin, calculated as silybin.

Adherence to silymarin supplementation was assessed at each scheduled outpatient visit during the 30-day treatment period by pill-count verification and inspection of empty or partially used blister packs. Treating physicians additionally conducted a structured adherence interview to confirm the dose intake and timing. When blister packs were not returned, the patients were specifically questioned regarding missed doses. Adherence was calculated as the proportion of pills consumed relative to the total number prescribed. All patients, including those with adherence <80%, were included in the intention-to-treat analysis, whereas per-protocol analyses excluded individuals below this adherence threshold.

### 2.8. Statistical Analysis

Statistical analyses were performed using MedCalc (v13.1.2). Multivariable regression analyses were additionally conducted using Python (version 3.13) with the statsmodels library. Continuous variables were presented as mean ± standard deviation or median (interquartile range), and categorical variables as counts and percentages. Between-group comparisons were conducted using Student’s *t*-test or Mann–Whitney U test for continuous variables and chi-square or Fisher’s exact test for categorical variables, as appropriate. Longitudinal changes were assessed using nonparametric repeated-measures analyses.

UACR constituted the principal renal outcome and was evaluated during the first three post-transplant months. Continuous UACR analyses were performed using available measurements at Months 1–3. The proportion of recipients achieving a ≥25% reduction in UACR between Months 1 and 3 was evaluated separately as an exploratory responder analysis and was restricted to recipients with paired UACR measurements at both time points. The responder threshold was defined as a ≥25% reduction in UACR from Month 1 to Month 3, in accordance with the threshold applied in a previous study [14]. Secondary outcomes were analyzed using complete-case data.

Multivariable analyses were performed using linear regression models (analysis of covariance, ANCOVA) for continuous outcomes and logistic regression models for binary outcomes. For UACR, log-transformed values were used because of the right-skewed distribution. An adjusted model including Month 1 UACR was used to characterize the subsequent association with Month 3 UACR; because Month 1 was a post-treatment assessment, this model was interpreted as a post-treatment trajectory analysis rather than as a conventional baseline-adjusted treatment-effect model. Similarly, eGFR and lipid parameters were analyzed as M3 values adjusted for Month 1 (M1) values. Exploratory clinically relevant categorical outcomes included a ≥25% reduction in UACR, ≥10% increase in eGFR, ≥15% reduction in LDL cholesterol or non-HDL cholesterol, and ≥15% increase in HDL cholesterol Covariates were selected a priori based on clinical relevance rather than automated statistical selection. Renal models included recipient age, donor characteristics, and peri-transplant variables, while metabolic models included body mass index, glycemic status, and immunosuppressive exposure. Sensitivity analyses additionally adjusted lipid models for initiation of lipid-lowering therapy during follow-up. To assess the potential impact of missing Month 3 UACR measurements, additional sensitivity analyses were performed. Characteristics of recipients with and without available Month 3 UACR were summarized descriptively within each cohort; because of the very small number of missing outcomes, no inferential statistical comparisons were performed. For the exploratory ≥25% UACR responder outcome, a conservative extreme-case analysis classified recipients with unavailable outcomes in the silymarin cohort as nonresponders and those with unavailable outcomes in the control cohort as responders. In addition, multiple imputation by chained equations was performed using 50 imputed datasets, incorporating available longitudinal UACR measurements and relevant clinical covariates. The adjusted continuous Month 3 UACR and responder models were then repeated across imputed datasets, with estimates combined using Rubin’s rules. The multiple-imputation analysis was considered a sensitivity analysis under a missing-at-random assumption conditional on the variables included in the imputation model.

Results were reported as regression coefficients or odds ratios with 95% confidence intervals. Statistical significance was defined as a two-sided *p* value < 0.05.

Detailed descriptions of the statistical methods, regression modeling strategies, missing data handling, and sample size calculations are provided in Supplementary Methods S1B.

## 3. Results

### 3.1. Participant Flow and Baseline Characteristics

**Figure 1:**
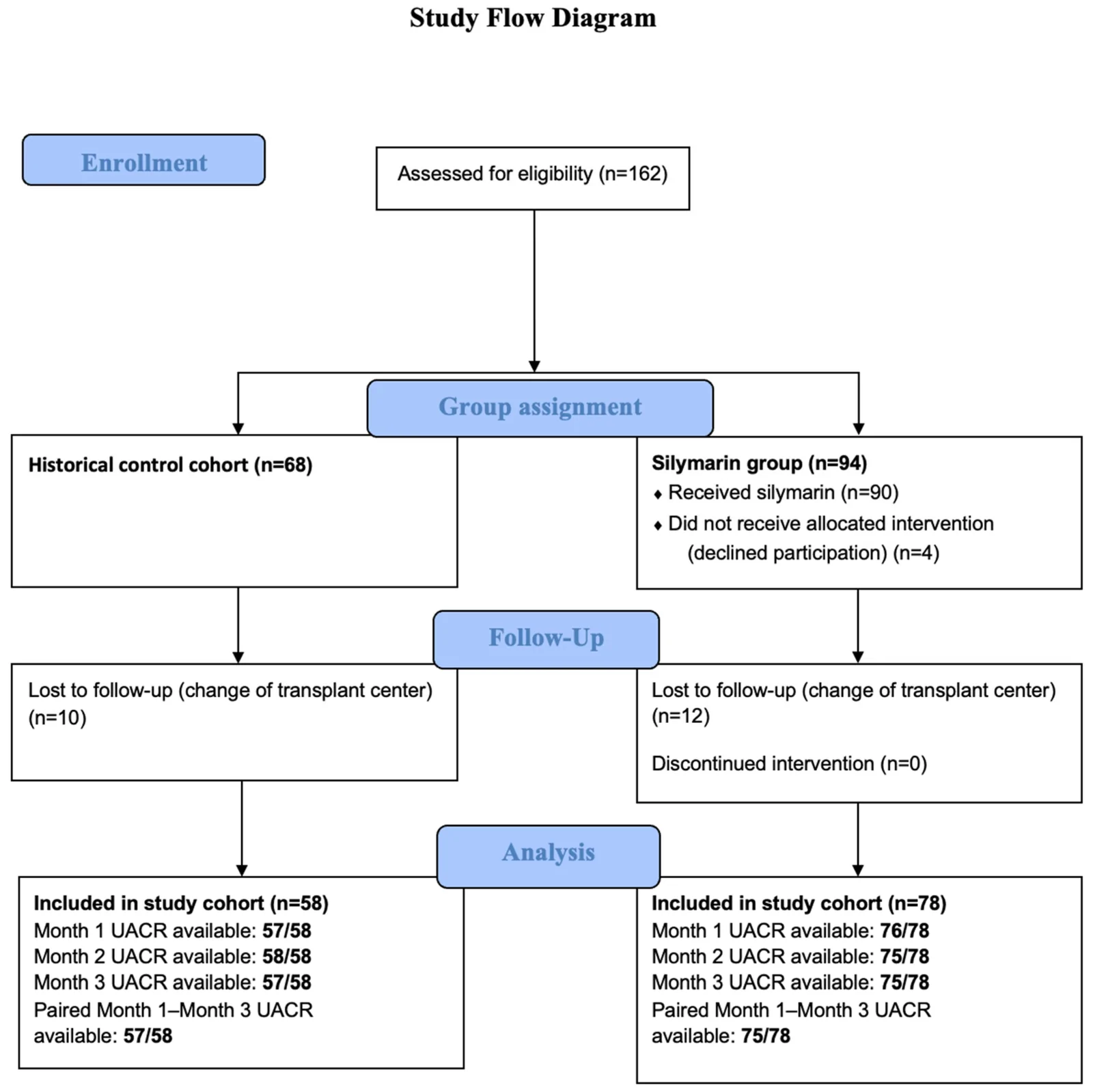
Study flow diagram. Flow diagram illustrating patient enrollment, allocation to control and silymarin-treated groups, follow-up, and inclusion in the final analyses. Missing UACR measurements reflected unavailable/not-collected urine measurements at the respective follow-up time points. No deaths or graft losses occurred during the 3-month follow-up.

Between January 2020 and April 2025, 162 kidney transplant procedures were performed at the Transplant-Nephrology Department. From January 2020 to September 2022, 68 recipients were identified for the historical control cohort; 10 did not complete the 3-month follow-up, resulting in 58 recipients included in the control cohort. From October 2022 to April 2025, 94 consecutive transplant recipients were screened for the prospective silymarin cohort; four declined participation and 12 did not complete the 3-month follow-up, resulting in 78 recipients included in the silymarin cohort. Overall, 136 recipients (78 silymarin-exposed and 58 historical controls) comprised the final study population. Availability of UACR measurements at individual follow-up time points and the numbers contributing to the paired Month 1–Month 3 analyses are summarized in Figure 1.

Baseline demographic and clinical characteristics are presented in Table 1 and Table 2.

Patients receiving silymarin were older than controls (49.3 ± 13.6 vs. 44.0 ± 16.1 years; *p* = 0.0380) and had a longer duration of pre-transplant dialysis (31.7 ± 32.0 vs. 26.2 ± 21.4 months; *p* = 0.0020). No significant differences were observed in sex distribution, body mass index, impaired glucose metabolism, cardiovascular or hepatic comorbidities, dialysis modality, or time on the transplant waiting list. Donor-related characteristics, including donor age, sex, donor type, cold ischemia time, and HLA mismatch profile, were comparable between groups. While overall sensitization rates were similar, class I DSA mean fluorescence intensity values were higher in the control cohort (11,663 ± 6915 vs. 2956 ± 3368; *p* = 0.0087). Class II DSA MFI values and prevalence of major histocompatibility complex (MHC) class I polypeptide-related sequence A antibodies did not differ between groups.

Induction immunosuppression regimens (basiliximab, ATG-F, or Thymoglobulin), cumulative ATG exposure, and the use of adjunct rituximab or intravenous immunoglobulins were similar across groups. The rates and timing of delayed graft function and early surgical complications did not differ. Detailed laboratory parameters across all post-transplant time points are provided in Supplementary Tables S1–S3.

### 3.2. Albuminuria Outcomes

UACR measurements were available in 76/78 (97.4%), 75/78 (96.2%), and 75/78 (96.2%) recipients in the silymarin cohort at Months 1, 2, and 3, respectively, and in 57/58 (98.3%), 58/58 (100%), and 57/58 (98.3%) recipients in the control cohort. Paired Month 1 and Month 3 UACR measurements were available in 75/78 (96.2%) and 57/58 (98.3%) recipients, respectively. Month 3 UACR was unavailable in 4/136 recipients (2.9%), including 3/78 (3.8%) in the silymarin cohort and 1/58 (1.7%) in the control cohort. No deaths or graft losses occurred during the 3-month follow-up. Characteristics of recipients with and without available Month 3 UACR are summarized descriptively in Supplementary Table S4. In unadjusted analyses, UACR values were significantly lower in the silymarin group at each monthly assessment. Importantly, the between-group difference was already present at Month 1, which represented the first standardized post-treatment UACR assessment and was obtained after completion of the 30-day silymarin exposure period. At M1, UACR was 12.86 ± 13.64 mg/mmol (median 8.1) versus 26.63 ± 25.45 mg/mmol (median 21.2) in controls (*p* < 0.0001). These differences persisted at M2 (7.01 ± 8.68 vs. 26.19 ± 50.51 mg/mmol; *p* < 0.0001) and M3 (7.04 ± 10.9 vs. 23.8 ± 44.7 mg/mmol; *p* < 0.0001) (Table 3).

Kidney function and albuminuria parameters were measured at predefined time points during the first three months after kidney transplantation in silymarin-treated and control recipients. Estimated glomerular filtration rate (eGFR) and urine albumin-to-creatinine ratio (UACR) are presented as mean ± standard deviation, with median values in parentheses. Between-group comparisons were performed at each time point using the appropriate statistical tests.

**Figure 2:**
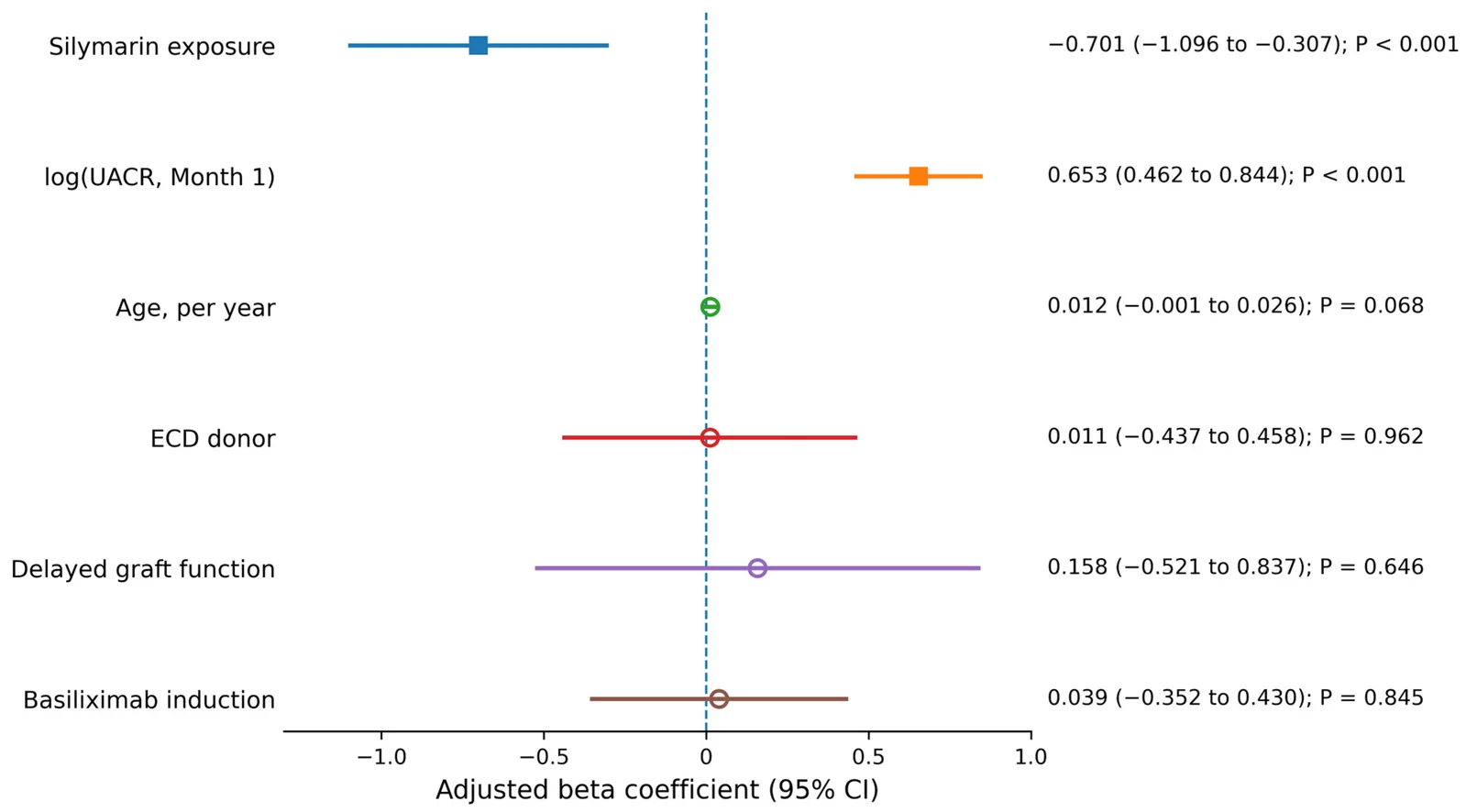
Forest plot of the multivariable linear regression analysis of log-transformed UACR at Month 3. UACR, urine albumin-to-creatinine ratio; M1, Month 1; M3, Month 3; ECD, expanded criteria donor; DGF, delayed graft function. Adjusted beta coefficients and 95% confidence intervals are shown for silymarin exposure, log-transformed Month 1 UACR, recipient age, expanded-criteria donor (ECD) status, delayed graft function (DGF), and basiliximab induction. Month 1 UACR represented the first standardized post-treatment assessment; therefore, the model characterizes the subsequent post-treatment UACR association rather than the total causal effect of silymarin exposure. Filled squares indicate statistically significant associations (*p* < 0.05), whereas open circles indicate non-significant associations. The vertical dashed line represents no association (β = 0).

Absolute changes in UACR between Month 1 and Month 2 and between Month 1 and Month 3 did not differ significantly between groups (median change M1–M2: −3.7 vs. −3.4 mg/mmol; *p* = 0.5488; M1–M3: −4.1 vs. −3.7 mg/mmol; *p* = 0.9817). In contrast, proportional changes differed significantly between groups, with greater median reductions in the silymarin cohort between Month 1 and Month 2 (−54.2% vs. −31.5%; *p* = 0.0391) and between Month 1 and Month 3 (−60.0% vs. −21.5%; *p* = 0.0396) (Supplementary Table S6). In the unadjusted analysis, silymarin exposure was associated with lower log-transformed UACR at Month 3 (β = −1.182, 95% CI −1.592 to −0.772; *p* < 0.001; n = 132). In a supportive multivariable analysis including Month 1 UACR and relevant clinical covariates, silymarin exposure remained associated with lower UACR at Month 3 (β = −0.701, 95% CI −1.096 to −0.307; *p* < 0.001; n = 132) (Table 4, Figure 2). Because Month 1 represented a post-treatment assessment, this model describes the subsequent Month 1-to-Month 3 association and does not estimate the total effect of silymarin exposure.

In a supportive longitudinal mixed-effects analysis of repeated UACR measurements from Month 1 to Month 3, the adjusted overall cohort-by-time interaction did not reach conventional statistical significance (likelihood-ratio χ^2^ = 5.59, 2 df; *p* = 0.061). UACR was already substantially lower in the silymarin cohort at Month 1 (adjusted geometric mean ratio [GMR] 0.41, 95% CI 0.28–0.59) and remained lower at Month 2 (GMR 0.29, 95% CI 0.20–0.42) and Month 3 (GMR 0.29, 95% CI 0.20–0.42). Thus, the longitudinal analysis demonstrated a persistent between-cohort difference from the first standardized post-treatment assessment onward, whereas the overall evidence for a differential subsequent Month 1-to-Month 3 trajectory did not reach statistical significance. Supportive longitudinal mixed-effects analyses are presented in Supplementary Table S5.

Linear regression models (analysis of covariance, ANCOVA) were constructed with log-transformed UACR at Month 3 as the dependent variable.

Models were adjusted for log-transformed UACR at Month 1, recipient age, expanded criteria donor (ECD) status, delayed graft function (DGF), and basiliximab induction. Log transformation was applied due to the right-skewed distribution of UACR.

Beta coefficients represent adjusted differences on the log scale.

In an exploratory responder analysis evaluating the achievement of a ≥25% reduction in UACR between M1 and M3, silymarin exposure was associated with higher odds of response (OR 3.28, 95% CI 1.38–7.79; *p* = 0.007) (Table 5).

Sensitivity analyses addressing missing Month 3 UACR yielded results broadly consistent with the primary adjusted analyses. In a conservative extreme-case analysis, in which recipients with unavailable responder outcomes in the silymarin cohort were classified as nonresponders and the corresponding recipient in the control cohort as a responder, response proportions were 46/78 (59.0%) and 28/58 (48.3%), respectively (unadjusted OR 1.54, 95% CI 0.78–3.05; Fisher’s exact *p* = 0.228). Multiple-imputation analysis using 50 imputed datasets yielded an adjusted association with Month 3 log-transformed UACR closely comparable to the primary complete-case model (β = −0.707, 95% CI −1.097 to −0.317; *p* < 0.001). The corresponding adjusted responder estimate was OR 3.37 (95% CI 1.42–8.02; *p* = 0.006).

No other clinical or treatment-related variables were consistently associated with UACR outcomes in multivariable models.

### 3.3. Graft Function

**Figure 3:**
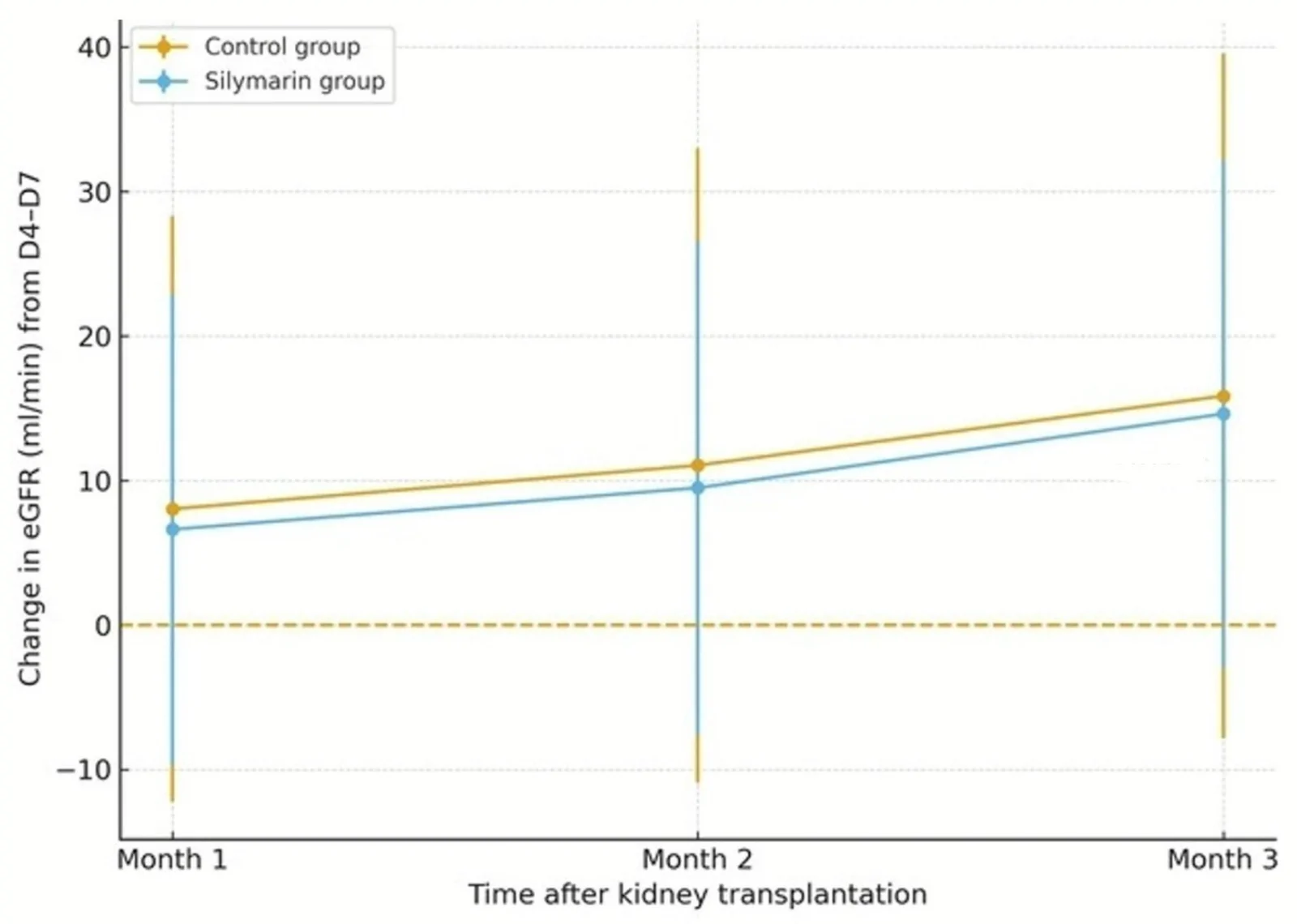
Longitudinal eGFR changes during the first 3 months after transplantation. Values represent mean change in estimated glomerular filtration rate from days 4–7 with 95% confidence intervals in the silymarin and control groups. Between-group differences at each time point were assessed using the Mann–Whitney U test. Dotted lines represent the 95% confidence intervals around the mean change. eGFR, estimated glomerular filtration rate.

Serial eGFR measurements during the first 3 months after transplantation are summarized in Table 3 and Figure 3.

Early post-transplant kidney function (days 4–7) did not differ between groups. A transient difference was observed at week 2, with higher eGFR in the control cohort (46.0 ± 24.2 vs. 37.2 ± 17.0 mL/min/1.73 m^2^; *p* = 0.0266) (Supplementary Table S1). However, this difference was no longer present at M1, M2, or M3.

Absolute and proportional changes in eGFR between M1 and M3 are shown in Supplementary Table S6. No significant between-group differences were observed.

In multivariable ANCOVA, silymarin exposure was not associated with eGFR at Month 3 (β = −0.53, 95% CI −5.29 to 4.24; *p* = 0.827), whereas Month 1 eGFR was strongly associated with Month 3 eGFR (β = 0.77, 95% CI 0.64–0.89; *p* < 0.001) (Table 6).

Similarly, silymarin exposure was not associated with the likelihood of achieving a ≥10% increase in eGFR between Month 1 and Month 3 (OR 1.35, 95% CI 0.60–3.03; *p* = 0.474). Higher Month 1 eGFR was associated with a lower likelihood of achieving this relative improvement (OR 0.95 per 1 mL/min/1.73 m^2^ increase, 95% CI 0.93–0.98; *p* < 0.001), while basiliximab induction was associated with a higher likelihood of achieving the ≥10% increase criterion (OR 2.98, 95% CI 1.13–7.87; *p* = 0.027) (Table 7).

Supportive longitudinal mixed-effects analysis showed no evidence of a differential eGFR trajectory between cohorts (adjusted cohort-by-time interaction: likelihood-ratio χ^2^ = 0.07, 2 df; *p* = 0.967) (Supplementary Table S5).

### 3.4. Lipid Profile

Lipid parameters at M1 and M3 are presented in Supplementary Table S7, with detailed longitudinal laboratory data provided in Supplementary Tables S1–S3. In unadjusted analyses, the control group exhibited higher levels of LDL cholesterol, non-HDL cholesterol, and total cholesterol from M1 onward, while HDL cholesterol was modestly higher in the control group during the early post-transplant period.

**Figure 4:**
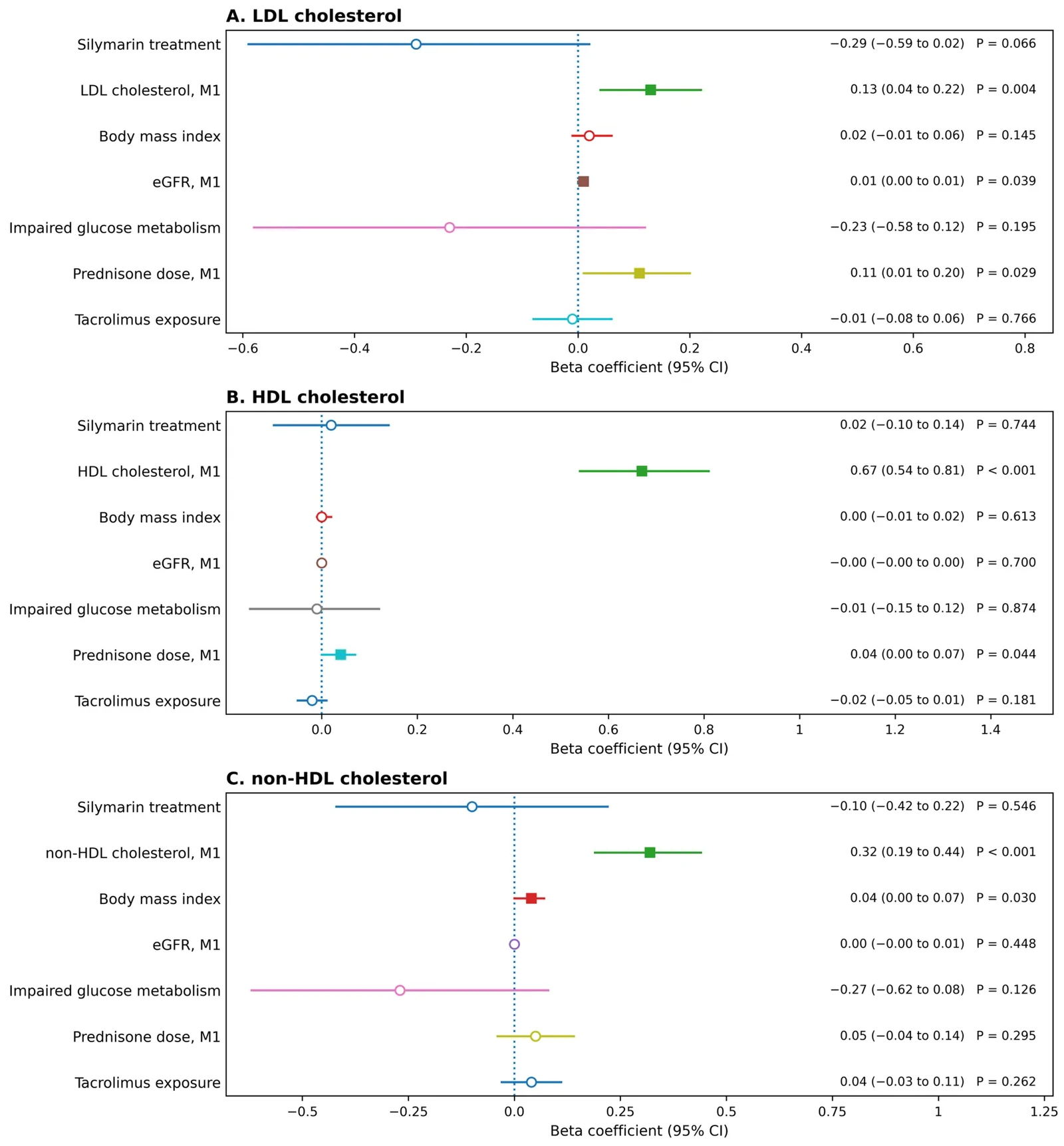
Forest plots of multivariable linear regression (ANCOVA) analysis of lipid parameters ((**A**): LDL, (**B**): HDL, (**C**): non-HDL). LDL, low-density lipoprotein; HDL, high-density lipoprotein; non-HDL, non-high-density lipoprotein; BMI, body mass index; eGFR, estimated glomerular filtration rate; M1, Month 1; M3, Month 3. Points represent adjusted beta coefficients and horizontal lines represent 95% confidence intervals. Filled squares indicate statistically significant associations (*p* < 0.05), whereas open circles indicate non-significant associations. The vertical blue dotted line represents no association (β = 0). Models were adjusted for the corresponding Month 1 lipid value, body mass index, eGFR at Month 1, impaired glucose metabolism, prednisone dose at Month 1, and tacrolimus exposure. LDL, low-density lipoprotein; HDL, high-density lipoprotein; non-HDL, non-high-density lipoprotein; BMI, body mass index; eGFR, estimated glomerular filtration rate; M1, Month 1; M3, Month 3.

In multivariable analysis, no consistent independent association between silymarin treatment and lipid parameters at M3 was observed after adjustment for Month 1 values and relevant covariates (Table 8, Figure 4).

Month 1 lipid levels were the strongest predictors of follow-up values across all models. Among covariates, prednisone exposure was associated with higher LDL and HDL cholesterol levels, while body mass index showed a modest association with non-HDL cholesterol.

Sensitivity analyses adjusting for initiation of lipid-lowering therapy yielded consistent results. Sensitivity analyses adjusting for initiation of lipid-lowering therapy yielded consistent results (Supplementary Table S8).

### 3.5. Glycaemic Parameters

The fasting and post-transplant glucose concentrations were comparable between the silymarin-treated and control groups at all time points, including days 4, 1, 2, and 1–3 (all *p* > 0.05). No significant differences were observed in the incidence of PTDM, impaired glucose level, or impaired fasting glucose (Supplementary Tables S1–S3).

At month 3, fasting C-peptide levels were similar between the silymarin-treated and control groups (4.6 ± 2.4 vs. 4.27 ± 2.24 ng/mL; *p* = 0.5361), as were 2-h postprandial C-peptide concentrations (13.8 ± 12.1 vs. 11.4 ± 5.94 ng/mL; *p* = 0.7418). Fasting insulin concentrations did not differ significantly (8.3 ± 6.8 vs. 10.1 ± 9.1 ng/mL; *p* = 0.0582), and 2-h postprandial insulin concentrations were likewise comparable (27.1 ± 17.8 vs. 45.6 ± 43.6 ng/mL; *p* = 0.1744). HbA1c measured at month 3 showed no between-group differences (4.2 ± 1.1% vs. 4.62 ± 1.57%; *p* = 0.1656) (Table 9).

Fasting and postprandial glucose metabolism parameters, indices of insulin secretion, and glycated hemoglobin were measured 3 months after transplantation in silymarin-treated and control recipients. Continuous variables are presented as mean ± standard deviation, with median values in parentheses and categorical variables as percentages. Between-group comparisons were performed using appropriate parametric or nonparametric tests. No statistically significant differences were observed between the groups.

In multivariable logistic regression analyses, silymarin therapy was not associated with the development of PTDM or prediabetic states. Fully adjusted models are provided in Supplementary Tables S9 and S10.

### 3.6. Immunologic Outcomes and Immunosuppression Exposure

Immunological outcomes during the first three months after transplantation are summarized in Table 10.

Immunologic events were infrequent and comparable between the groups. Antibody-mediated rejection was observed only in the control cohort. At M3, donor-specific HLA antibodies were uncommon in both groups, with no relevant differences in antibody class or signal intensity. MHC class I polypeptide-related sequence A antibodies were rare.

Maintenance immunosuppression was comparable across cohorts throughout follow-up. Tacrolimus-based regimens, mycophenolic acid, and corticosteroids were used in the majority of recipients, with similar tacrolimus exposure and steroid dosing. Minor differences in mycophenolic acid dosing during early follow-up did not translate into meaningful differences in overall immunosuppressive exposure.

### 3.7. Safety Outcomes and Adverse Events

No adverse events attributable to silymarin supplementation were reported. Self-reported adherence to the 30-day regimen was 100%, and no patient reported discontinuation or missed dose.

Liver enzyme measurements showed no significant between-group differences in aspartate aminotransferase, alanine aminotransferase, or alkaline phosphatase levels at any time point. Gamma-glutamyl transferase levels were higher in the control group at month 1 (1.42 ± 1.35 vs. 0.95 ± 0.86 µkat/L; *p* = 0.0040) and month 2 (0.97 ± 1.09 vs. 0.78 ± 1.34 µkat/L; *p* = 0.0159), while no differences were observed at day 4 or month 3 (Supplementary Tables S1–S3).

## 4. Discussion

In this prospective cohort study of kidney transplant recipients, early short-term silymarin supplementation was associated with a higher likelihood of achieving a clinically meaningful reduction in albuminuria during the first three months after transplantation. Despite comparable baseline characteristics and standardized immunosuppressive protocols, patients receiving silymarin demonstrated lower UACR values throughout the first three post-transplant months, while graft function recovery assessed by eGFR, lipid parameters, glycemic outcomes, and immunological events remained largely unaffected. However, the UACR difference was already present at Month 1, the first standardized post-transplant assessment, which was obtained after completion of the 30-day silymarin exposure period. Therefore, the present study cannot determine whether this early difference resulted from silymarin exposure or from residual between-cohort differences, calendar-time effects, early graft recovery, selection, or other unmeasured factors. These findings address a clinically relevant gap, as prospective data on the early post-transplant effects of silymarin are limited, and suggest that silymarin may exert a selective protective effect on the glomerular filtration barrier rather than on global graft function or systemic metabolic parameters.

The observed reduction in albuminuria is biologically plausible and consistent with the proposed mechanism of action of silymarin. Ischemia–reperfusion injury, an inevitable consequence of kidney transplantation, induces oxidative stress, lipid peroxidation, endothelial dysfunction, and inflammatory activation, all of which contribute to disruption of the glomerular filtration barrier. Experimental and preclinical studies have demonstrated that silymarin attenuates these pathways through suppression of lipid peroxidation, reduction of MDA and TNF-α, and downregulation of endothelial adhesion molecules including vascular cell adhesion molecule-1 and intercellular adhesion molecule-1, thereby limiting inflammatory cell recruitment and secondary tissue injury [1,2,3,7]. Furthermore, Fallahzadeh et al. demonstrated concomitant reductions in urinary albumin excretion, MDA, and TNF-α following silymarin supplementation in patients with diabetic nephropathy [8]. Although these biomarkers were not directly measured in the present study, our findings are compatible with the hypothesis that attenuation of oxidative stress and inflammation may preserve the integrity of the glomerular filtration barrier during the early post-transplant period.

The consistency of lower UACR values across Months 1–3 supports the robustness of the observed between-cohort association; however, it does not resolve the uncertainty regarding the origin of the difference already present at Month 1. In addition to lower UACR values observed at all post-transplant time points, silymarin-treated patients demonstrated greater proportional reductions in albuminuria despite comparable absolute changes. However, supportive longitudinal mixed-effects analysis showed that UACR was already substantially lower in the silymarin cohort at Month 1, and the overall cohort-by-time interaction from Month 1 to Month 3 did not reach conventional statistical significance. Accordingly, the findings primarily indicate a persistent between-cohort difference in UACR from the first standardized post-treatment assessment onward, rather than definitive evidence of a distinct subsequent longitudinal treatment trajectory. In the endpoint-specific adjusted analysis, silymarin exposure remained associated with lower UACR at Month 3 after adjustment for Month 1 UACR and relevant clinical covariates. The association remained evident in adjusted analyses; however, because Month 1 UACR was a post-treatment measurement, these models should be interpreted as describing the subsequent post-treatment association rather than estimating change from a pretreatment baseline. Consistent with this, an exploratory responder analysis showed higher odds of achieving a ≥25% reduction in UACR between Months 1 and 3 among silymarin-exposed recipients.

Clinical data on the effects of silymarin on albuminuria in kidney transplant recipients remain scarce. The most robust evidence originates from a randomized, double-blind, placebo-controlled trial in patients with type 2 diabetes and overt diabetic nephropathy, in which adjunctive silymarin therapy combined with renin–angiotensin system inhibition resulted in a significantly greater reduction in UACR, accompanied by decreases in inflammatory, oxidative stress, and profibrotic markers including tumor necrosis factor-α and transforming growth factor-β [8]. Given the well-established dose-dependent association between post-transplant proteinuria and long-term graft failure, attenuation of early albuminuria may have important prognostic implications, although confirmation requires longer follow-up and randomized clinical studies [15].

In contrast to the consistent effects on albuminuria, silymarin supplementation was not associated with measurable improvement in estimated glomerular filtration rate during the first three months after transplantation. Early graft function was comparable between groups, and the transient difference observed at week 2 was not sustained beyond the first month. This finding is not unexpected, as early post-transplant eGFR is influenced by numerous competing factors, including donor characteristics, ischemia–reperfusion injury, delayed graft function, perioperative hemodynamics, calcineurin inhibitor exposure, rejection episodes, and fluid status [16]. Moreover, albuminuria and eGFR reflect distinct biological processes. While eGFR represents overall filtration capacity, albuminuria is a more sensitive marker of glomerular permeability, endothelial integrity, and inflammatory injury [17]. Therefore, preservation of glomerular filtration barrier integrity may precede measurable changes in global filtration capacity, explaining why improvements in albuminuria can occur in the absence of early differences in eGFR. This dissociation has been described in both native and transplanted kidney disease and supports the interpretation that the albuminuria-lowering effect observed in the SAGE study may reflect a targeted renoprotective effect on the glomerular filtration barrier rather than an early improvement in overall graft filtration.

In contrast to the findings on albuminuria, the effects of silymarin on lipid parameters were not consistently confirmed after adjustment for M1 values and relevant covariates. Although unadjusted analyses suggested lower concentrations of total cholesterol, LDL cholesterol, and non-HDL cholesterol during the early post-transplant period, these differences were not independently associated with silymarin treatment in multivariable models. Month 1 lipid levels remained the strongest predictors of follow-up values, indicating that the observed unadjusted differences were likely influenced by early post-transplant variability rather than a direct treatment effect.

Nevertheless, a modest signal toward LDL cholesterol modulation was observed, which is in line with previously reported lipid-lowering effects of silymarin in non-transplant populations [18]. Similarly, in our previous pilot study of stable kidney transplant recipients receiving six months of silymarin supplementation, we observed a significant reduction in LDL cholesterol among patients with diabetes or prediabetes, although no significant effects on graft function or albuminuria were detected [19]. This discrepancy is likely explained by substantial differences in study design, patient population, treatment duration, and primary objectives between the two studies. While the previous pilot study focused on long-term metabolic effects in stable transplant recipients without a control group, the present study evaluated early post-transplant supplementation and its association with albuminuria in a controlled cohort. Therefore, the present findings should be interpreted as complementary rather than confirmatory. Given the high burden of cardiovascular risk in kidney transplant recipients, even subtle modulation of LDL cholesterol warrants further investigation in adequately powered randomized studies.

No significant effects of silymarin on glucose metabolism were observed. Although randomized trials and meta-analyses in non-transplant populations have reported improvements in fasting glucose, insulin resistance, and glycated hemoglobin, particularly among patients with established insulin resistance or type 2 diabetes mellitus, these findings were not reproduced in our cohort. Similarly, our previous pilot study in stable kidney transplant recipients did not demonstrate significant improvements in glucose metabolism following six months of silymarin supplementation. This discrepancy may reflect the short duration of supplementation, the relatively low number of glycemic events, and the profound diabetogenic milieu of the early post-transplant period, characterized by corticosteroid therapy, calcineurin inhibitor exposure, surgical stress, and systemic inflammation.

Silymarin supplementation was well tolerated, with no adverse events attributable to the intervention. Liver enzyme profiles remained comparable between groups, with no evidence of hepatocellular or cholestatic toxicity. The transiently lower gamma-glutamyl transferase levels observed in the silymarin group during the early post-transplant period may reflect reduced oxidative or metabolic hepatic stress rather than a direct pharmacologic effect. Importantly, no interactions with calcineurin inhibitors or other components of standard immunosuppressive regimens were observed, consistent with previous safety data [18,20,21]. Taken together, these findings indicate that the albuminuria-lowering effect of silymarin was achieved without compromising safety or immunosuppressive management.

This study has several limitations. A major limitation is the absence of a standardized pretreatment UACR measurement. The first standardized UACR assessment was obtained at Month 1, after completion of the 30-day silymarin exposure period, and UACR was already lower in the silymarin cohort at that time. Consequently, the study cannot determine whether this difference developed during silymarin exposure or reflected pre-existing differences between cohorts, calendar-time effects, variation in early graft recovery, contribution from native kidneys, selection, or other unmeasured confounding. Furthermore, analyses incorporating Month 1 UACR condition on a measurement obtained after initiation of silymarin exposure and therefore potentially on a treatment-affected variable. Accordingly, these analyses were interpreted as characterizing the subsequent post-treatment UACR trajectory rather than estimating the total causal effect of silymarin exposure. Because cohort assignment was determined by transplantation period, residual calendar-time confounding cannot be completely excluded despite unchanged donor selection practices, institutional clinical protocols, laboratory methods, and follow-up procedures. Follow-up was limited to three months, precluding assessment of long-term graft and cardiovascular outcomes. Although mean tacrolimus trough concentrations did not differ significantly between groups, intra-individual tacrolimus variability, dose-normalized exposure, and formal pharmacokinetic interactions were not evaluated. Given the narrow therapeutic index of tacrolimus and the limited clinical evidence regarding concomitant administration with silymarin, a clinically relevant interaction cannot be excluded, and dedicated pharmacokinetic and safety studies are warranted. Month 3 UACR was unavailable in 2.9% of recipients. Although the small number of missing outcomes limits formal assessment of the missing-data mechanism and residual attrition bias cannot be completely excluded, multiple-imputation analyses yielded effect estimates closely comparable to the primary complete-case analyses. No deaths or graft losses occurred among recipients with unavailable Month 3 UACR. The observational design precludes causal inference, and some secondary analyses were limited by statistical power. In addition, oxidative stress and inflammatory biomarkers, including MDA and TNF-α, were not measured directly. Consequently, the proposed biological mechanism underlying the observed albuminuria association remains inferential and warrants confirmation in future mechanistic studies. Despite these limitations, the present study has several important strengths. It provides prospective, real-world data on early post-transplant dynamics in a well-characterized cohort managed within a uniform clinical protocol, including standardized immunosuppression, follow-up schedules, and comprehensive metabolic assessment. The focus on the immediate post-transplant period, a critical yet understudied window, enabled evaluation of early modifiable risk markers with potential long-term prognostic relevance. Importantly, this study is among the first to systematically assess the effect of silymarin on albuminuria in kidney transplant recipients, thereby addressing a clinically relevant gap and providing novel evidence for a targeted effect on early glomerular injury.

Future randomized, placebo-controlled, multicenter trials with longer follow-up are required to determine whether early attenuation of albuminuria translates into improved long-term graft and cardiovascular outcomes. Incorporation of serial measurements of oxidative stress and inflammatory biomarkers, together with protocol biopsy findings, may further clarify whether the observed clinical effects are mediated through attenuation of ischemia–reperfusion injury and preservation of the glomerular filtration barrier.

## 5. Conclusions

In conclusion, early short-term silymarin exposure was associated with lower UACR during the first three months after kidney transplantation, without a measurable effect on estimated glomerular filtration rate, immunological outcomes, or metabolic parameters. Because the between-group difference in UACR was already present at Month 1 and no pretreatment UACR measurement was available, these findings should be interpreted as associative rather than causal. This effect was observed across complementary analytical approaches and supports the concept of a targeted influence on early glomerular injury rather than a global improvement in filtration function. Importantly, silymarin was well tolerated and did not interfere with standard immunosuppressive therapy.

Given the established link between early albuminuria and long-term graft and cardiovascular outcomes, these findings suggest that short-term silymarin therapy may represent a safe and accessible adjunctive strategy to modulate early post-transplant risk. Confirmation in randomized studies with longer follow-up is warranted to determine whether these early effects translate into sustained clinical benefit.

## Figures and Tables

**Table 1:** Baseline Characteristics of the Study Population.

Parameter	Silymarin Group (n = 78)	Control Group (n = 58)	*p* Value
Age (years)	49.3 ± 13.6	44.0 ± 16.1	**0.0380**
Sex, male (%)	48 (61.5%)	37 (63.8%)	0.9461
Body mass index (kg/m^2^)	26.1 ± 4.4	26.5 ± 5.1	0.4475
History of impaired glucose metabolism (%)	19 (24.4%)	15 (25.9%)	0.8431
Type 1 diabetes	8 (10.3%) [42.1% of DM]	6 (10.3%) [40% of DM]	0.9867
Type 2 diabetes	7 (9.0%) [36.8% of DM]	6 (10.3%) [40% of DM]	0.7888
Impaired glucose tolerance	3 (3.8%) [15.8% of DM]	3 (5.2%) [20% of DM]	0.7106
Impaired fasting glucose	1 (1.3%) [5.3% of DM]	0% (0)	0.3885
Arterial hypertension	76 (97.4%)	56 (96.6%)	0.7636
Liver disease	19 (24.4%)	14 (24.1%)	0.9764
Cardiovascular disease	11 (14.1%)	8 (13.8%)	0.9591
Coronary heart disease	5 (6.4%) [45.5% of CVD]	3 (5.2%) [37.5% of CVD]	0.7624
Atrial fibrillation	5 (6.4%) [45.5% of CVD]	1 (1.7%) [12.5% of CVD]	0.0833
Hypertensive heart disease	1 (1.3%) [9.1% of CVD]	2 (3.4%) [25% of CVD]	0.3122
Other	0 (0%)	2 (3.4%) [25% of CVD]	0.1800
Renal replacement therapy			0.1718
HD	69 (88.5%)	48 (82.8%)	
PD	4 (5.1%)	8 (13.8%)	
preemptive	5 (6.4%)	2 (3.4%)	
Duration of dialysis (months)	31.7 ± 32	26.2 ± 21.4	**0.0020**
Waiting-list duration (days)	405 ± 516	379 ± 487	0.6530

DM—diabetes mellitus, CVD—cardiovascular disease, HD—hemodialysis, PD—peritoneal dialysis. Baseline demographic and clinical characteristics of study population. The recipient demographics, comorbidities, glucose metabolism status, renal replacement therapy, and transplant-related variables were summarized for the silymarin-treated and control groups. Data are presented as mean ± standard deviation for approximately normally distributed continuous variables, and n (%) for categorical variables. Between-group comparisons were performed using Student’s *t*-test or the Mann–Whitney U test for continuous variables and the chi-square test or Fisher’s exact test for categorical variables, as appropriate. For multi-category variables, the reported *p* value represents the overall between-group comparison. Statistically significant *p* values (*p* < 0.05) are shown in bold.

**Table 2:** Donor and Transplantation Characteristics.

Parameter	Silymarin Group (n = 78)	Control Group (n = 58)	*p* Value
Type of donor			0.5210
SCD	44 (56.4%)	38 (65.5%)	
ECD	22 (28.2%)	14 (24.1%)	
living donor	12 (15.4%)	6 (10.3%)	
Donor age (years)	49.6 ± 13.4	46.2 ± 13.2	0.1508
Donor sex, male	39 (50%)	33 (56.9%)	0.4885
Cold ischemia time (minutes)	651 ± 331	556 ± 306	0.0909
HLA mismatch—A	1(1–1)	1 (1–2)	0.8902
HLA mismatch—B	1 (1–2)	1 (1–2)	0.1888
HLA mismatch—DR	1 (1–1)	1 (0–1)	0.3500
Panel reactive antibodies—actual	1 (0–3)	0 (0–2)	0.2449
Panel reactive antibodies—maximum	1 (0–5.5)	1 (0–4.5)	0.0824
Donor-specific antibody positive	16 (20.5%)	8 (13.8%)	0.3676
Class I DSA MFI	1410 (725–3879.5)	11,950 (5300–18,000)	**0.0087**
Class II DSA MFI	2789 (1460–14,600)	4000 (3200–12,300)	0.7028
MICA antibodies positive	7 (9%)	3 (5.2%)	0.5160
Order of transplantation			0.6615
primary	71 (91%)	54 (93.1%)	
secondary	7 (9%)	4 (6.9%)	
Induction therapy			0.0901
basiliximab	22 (28.2%)	25 (43.1%)	
ATG-F	19 (24.4%)	7 (12.1%)	
ATG	37 (47.4%)	26 (44.8%)	
Cumulative ATG dose (mg/kg)	3.5 (3.5–5.0)	3.5 (3.5–3.5)	0.0777
Rituximab added	2 (2.6%)	1 (1.7%)	0.7424
IVIG added	1 (1.3%)	1 (1.7%)	0.8329
Delayed graft function	8 (10.3%)	4 (6.9%) (4)	0.557
Surgical complication after KT	9 (11.5%)	13 (22.4%)	0.103
Time to complication (weeks)	1 (1–1)	1 (1–1)	0.3840

ATG, anti-thymocyte globulin; ATG-F, antithymocyte globulin-F/Grafalon; DSA, donor-specific antibody; ECD, expanded criteria donor; HLA, human leukocyte antigen; IVIG, intravenous immunoglobulins; KT, kidney transplantation; MFI, mean fluorescence intensity; MICA, major histocompatibility complex class I polypeptide-related sequence A; SCD, standard criteria donor. Data are presented as mean ± standard deviation for approximately normally distributed continuous variables, median (Q1–Q3) for skewed or ordinal variables, and n (%) for categorical variables. Between-group comparisons were performed using Student’s *t*-test for approximately normally distributed continuous variables, the Mann–Whitney U test for skewed or ordinal variables, and the chi-square test or Fisher’s exact test for categorical variables, as appropriate. For multi-category variables, the reported *p* value represents the overall between-group comparison. Statistically significant *p* values (*p* < 0.05) are shown in bold. Note: The Cumulative ATG-Fresenius dose was identical in all patients (11 mg/kg). ATG = Thymoglobuline.

**Table 3:** Kidney Function and Albuminuria During the First 3 Months After Kidney Transplantation.

Time Point	Parameter	Silymarin Group (n = 78)	Control Group (n = 58)	*p* Value
Day 4	eGFR (mL/min/1.73 m^2^)	27.7 ± 17.0	36.0 ± 26.0	0.0912
Week 1	eGFR (mL/min/1.73 m^2^)	37.0 ± 20.3	46.0 ± 31.4	0.1989
Week 2	eGFR (mL/min/1.73 m^2^)	37.2 ± 17.0	46.0 ± 24.2	**0.0266**
Month 1	eGFR (mL/min/1.73 m^2^)	43.6 ± 17.2	49.1 ± 24.1	0.1266
Month 1	UACR (mg/mmol)	8.1 (4.1–15.5)	21.7 (10.7–31.2)	**<0.0001**
Month 2	eGFR (mL/min/1.73 m^2^)	46.0 ± 16.0	52.1 ± 26.1	0.2326
Month 2	UACR (mg/mmol)	3.8 (1.8–8.4)	13.1 (7.0–26.5)	**<0.0001**
Month 3	eGFR (mL/min/1.73 m^2^)	51.3 ± 15.4	57.4 ± 27.2	0.1007
Month 3	UACR (mg/mmol)	3.7 (1.6–7.1)	13.8 (7.8–24.6)	**<0.0001**

eGFR, estimated glomerular filtration rate; UACR, urine albumin-to-creatinine ratio. Available UACR measurements were: Month 1, 76/78 silymarin and 57/58 control; Month 2, 75/78 and 58/58; Month 3, 75/78 and 57/58, respectively. Data are presented as mean ± standard deviation for approximately normally distributed variables and median (Q1–Q3) for skewed variables. Between-group comparisons were performed using Student’s *t*-test for approximately normally distributed variables and the Mann–Whitney U test for skewed variables, as appropriate. Analyses were based on available observations at each time point. Statistically significant *p* values (*p* < 0.05) are shown in bold.

**Table 4:** Adjusted linear regression model of log-transformed UACR at Month 3 (n = 132).

Variable	Beta	95% CI	*p* Value
Silymarin treatment	−0.701	−1.096 to −0.307	**<0.001**
log(UACR, Month 1)	0.653	0.462–0.844	**<0.001**
Age, per year	0.012	−0.001–0.026	0.068
ECD	0.011	−0.437–0.458	0.962
Delayed graft function	0.158	−0.521–0.837	0.646
Basiliximab induction	0.039	−0.352–0.430	0.845

CI, confidence interval; UACR, urine albumin-to-creatinine ratio; ECD, expanded criteria donor; DGF, delayed graft function. Beta coefficients represent adjusted differences in log-transformed UACR at Month 3. The multivariable model included silymarin exposure, log-transformed Month 1 UACR, recipient age, expanded criteria donor (ECD) status, delayed graft function (DGF), and basiliximab induction. Month 1 UACR represented the first standardized post-treatment assessment and was not a pretreatment baseline. Accordingly, the model characterizes the subsequent Month 1-to-Month 3 UACR association and should not be interpreted as estimating the total causal effect of silymarin exposure. Statistically significant *p* values (*p* < 0.05) are shown in bold.

**Table 5:** Exploratory multivariable logistic regression analysis of ≥25% UACR reduction between Month 1 and Month 3.

Variable	OR	95% CI	*p* Value
Silymarin treatment	3.28	1.38–7.79	**0.007**
log(UACR, Month 1)	1.75	1.15–2.66	**0.009**
Age, per year	0.97	0.95–1.00	0.067
ECD	1.34	0.52–3.47	0.544
Delayed graft function	1.68	0.37–7.59	0.499
Basiliximab induction	0.91	0.40–2.05	0.820

OR, odds ratio; CI, confidence interval; UACR, urine albumin-to-creatinine ratio; ECD, expanded criteria donor; DGF, delayed graft function. Odds ratios represent the adjusted likelihood of achieving a ≥25% reduction in UACR between Month 1 and Month 3 among recipients with paired UACR measurements at both time points (n = 132; 75/78 in the silymarin cohort and 57/58 in the control cohort). The multivariable logistic regression model included log-transformed Month 1 UACR, recipient age, expanded criteria donor (ECD) status, delayed graft function (DGF), and basiliximab induction. Month 1 UACR was log-transformed because of its right-skewed distribution. Because Month 1 UACR was measured after the 30-day exposure period, the responder outcome reflects the subsequent post-treatment UACR trajectory and should not be interpreted as change from a pretreatment baseline or as an estimate of the total treatment effect. Statistically significant *p* values (*p* < 0.05) are shown in bold.

**Table 6:** Multivariable linear regression (ANCOVA) analysis of eGFR at Month 3.

Variable	Beta	95% CI	*p* Value
Silymarin treatment	−0.53	−5.29–4.24	0.827
eGFR at Month 1	0.77	0.64–0.89	**<0.001**
Age	−0.11	−0.29–0.07	0.215
ECD	−2.33	−8.37–3.70	0.446
Delayed graft function	2.36	−6.87–11.59	0.614
Basiliximab induction	4.14	−1.08–9.36	0.119

CI, confidence interval; eGFR, estimated glomerular filtration rate; ECD, expanded criteria donor; DGF, delayed graft function. Linear regression models (analysis of covariance, ANCOVA) were constructed with eGFR at Month 3 as the dependent variable. Models were adjusted for eGFR at Month 1, recipient age, expanded criteria donor (ECD) status, delayed graft function (DGF), and basiliximab induction. Beta coefficients represent adjusted differences in eGFR (mL/min/1.73 m^2^). Statistically significant *p* values (*p* < 0.05) are shown in bold.

**Table 7:** Multivariable logistic regression analysis for ≥10% increase in eGFR between Month 1 and Month 3.

Variable	OR	95% CI	*p* Value
Silymarin treatment	1.35	0.60–3.03	0.474
eGFR at Month 1	0.95	0.93–0.98	**<0.001**
Age	0.99	0.96–1.02	0.582
ECD	0.56	0.21–1.52	0.255
Delayed graft function	0.92	0.15–5.57	0.931
Basiliximab induction	2.98	1.13–7.87	**0.027**

CI, confidence interval; eGFR, estimated glomerular filtration rate; ECD, expanded criteria donor; DGF, delayed graft function. Odds ratios (ORs) represent the adjusted likelihood of achieving a ≥10% increase in eGFR between Month 1 and Month 3. Models were adjusted for eGFR at Month 1, recipient age, expanded criteria donor (ECD) status, delayed graft function (DGF), and basiliximab induction. Statistically significant *p* values (*p* < 0.05) are shown in bold.

**Table 8:** Multivariable linear regression (ANCOVA) analysis of lipid parameters at Month 3.

Outcome	Variable	β	95% CI	*p* Value
LDL cholesterol	Silymarin treatment	−0.29	−0.59 to 0.02	0.066
	LDL cholesterol at Month 1	0.13	0.04 to 0.22	**0.004**
	Body mass index	0.02	−0.01 to 0.06	0.145
	eGFR at Month 1	0.01	0.00 to 0.01	**0.039**
	Impaired glucose metabolism	−0.23	−0.58 to 0.12	0.195
	Prednisone dose at Month 1	0.11	0.01 to 0.20	**0.029**
	Tacrolimus mean level	−0.01	−0.08 to 0.06	0.766
HDL cholesterol	Silymarin treatment	0.02	−0.10 to 0.14	0.744
	HDL cholesterol at Month 1	0.67	0.54 to 0.81	**<0.001**
	Body mass index	0.00	−0.01 to 0.02	0.613
	eGFR at Month 1	−0.00	−0.00 to 0.00	0.700
	Impaired glucose metabolism	−0.01	−0.15 to 0.12	0.874
	Prednisone dose at Month 1	0.04	0.00 to 0.07	**0.044**
	Tacrolimus mean level	−0.02	−0.05 to 0.01	0.181
Non-HDL cholesterol	Silymarin treatment	−0.10	−0.42 to 0.22	0.546
	Non-HDL cholesterol at Month 1	0.32	0.19 to 0.44	**<0.001**
	Body mass index	0.04	0.00 to 0.07	**0.030**
	eGFR at Month 1	0.00	−0.00 to 0.01	0.448
	Impaired glucose metabolism	−0.27	−0.62 to 0.08	0.126
	Prednisone dose at Month 1	0.05	−0.04 to 0.14	0.295
	Tacrolimus mean level	0.04	−0.03 to 0.11	0.262

CI, confidence interval; eGFR, estimated glomerular filtration rate; M1, Month 1; M3, Month 3; LDL, low-density lipoprotein; HDL, high-density lipoprotein. Multivariable linear regression models (analysis of covariance, ANCOVA) were constructed with lipid parameters at Month 3 as dependent variables. Each model was adjusted for the corresponding lipid value at Month 1, body mass index, estimated glomerular filtration rate at Month 1, impaired glucose metabolism, prednisone dose at Month 1, and tacrolimus exposure. Beta coefficients represent adjusted differences in lipid parameters (mmol/L). Statistically significant *p* values (*p* < 0.05) are shown in bold.

**Table 9:** Glycaemic Parameters at Month 3 after transplantation.

Parameter	Silymarin (n = 78)	Control (n = 58)	*p* Value
Fasting glucose (mmol/L)	6.01 ± 1.92	5.75 ± 1.87	0.7198
Fasting insulin (mU/L)	8.3 ± 6.8	10.1 ± 9.1	0.0582
2 h insulin (mU/L)	27.1 ± 17.8	45.6 ± 43.6	0.1744
Fasting C-peptide (ng/mL)	4.6 ± 2.4	4.27 ± 2.24	0.5361
2 h C-peptide (ng/mL)	13.8 ± 12.1	11.4 ± 5.94	0.7418
HbA1c (%)	4.2 ± 1.1	4.62 ± 1.57	0.1656
PTDM (%)	9 (11.5%)	8 (13.8%)	0.4349
IGT (%)	15 (19.2%)	5 (8.6%)	0.1407
IFG (%)	0 (0%)	1 (1.7%)	0.2462

2 h C-peptide, 2-h postprandial C-peptide; 2 h insulin, 2-h postprandial insulin; Fasting C-peptide, fasting C-peptide concentration; Fasting glucose, fasting plasma glucose; Fasting insulin, fasting plasma insulin; HbA1c, glycated hemoglobin; IFG, impaired fasting glucose; IGT, impaired glucose tolerance; PTDM, post-transplant diabetes mellitus.

**Table 10:** Immunologic Outcomes & Immunosuppression.

Parameter	Silymarin	Control	*p* Value
TCMR (%)	3 (3.8%)	5 (8.6%)	0.3300
ABMR (%)	0 (0%)	3 (5.2%)	0.0890
DSA positive	4 (5.1%)	6 (10.3%)	0.3242
Class I MFI	2445 ± 1841	2693 ± 3000	1.000
Class II MFI	4380 ± 4780	2700 ± 2700	0.800
MICA antibodies	1 (1.3%)	1 (1.7%)	0.8329
Tacrolimus trough			
Month 1	14.1 ± 3.2	15.5 ± 3.5	0.1067
Month 2	13.6 ± 3.29	13.4 ± 3.8	0.4364
Month 3	10.7 ± 2.35	10.6 ± 2.6	0.3176
MPA dose (mg/day)			
Month 1	1028	1213	**<0.0001**
Month 2	935	1021	**0.0333**
Month 3	842	871	0.5359
Prednisone dose (mg/day)			
Month 1	14.4	14.6	0.5893
Month 2	10.1	9.9	0.1547
Month 3	10.4	9.9	0.7636

ABMR, antibody-mediated rejection; DSA, donor-specific antibodies; KT, kidney transplantation; MFI, mean fluorescence intensity; MPA, mycophenolic acid; MICA, major histocompatibility complex class I polypeptide-related sequence A; TCMR, T-cell-mediated rejection. Immunologic outcomes and immunosuppressive exposure during the first 3 months after kidney transplantation in silymarin-treated and control recipients. Acute rejection phenotypes, donor-specific antibodies, and maintenance immunosuppression parameters were summarized. Continuous variables are presented as the mean ± standard deviation, and categorical variables are presented as percentages with absolute counts in parentheses. Between-group comparisons were performed using appropriate statistical tests.

## Data Availability

Anonymized data that support the findings of this study are available from the corresponding author upon reasonable request.

## Supplementary Materials

The following supporting information can be downloaded at: https://www.mdpi.com/article/10.3390/jcm15176694/s1, Supplementary Methods S1A. Materials and Methods. Supplementary Methods S1B. Statistical analysis. Supplementary Table S1. Laboratory parameters from the day of kidney transplantation to week 2 after transplantation. Supplementary Table S2. Detailed laboratory parameters at month 1 and 2 after kidney transplantation. Supplementary Table S3. Detailed laboratory parameters at month 3 after kidney transplantation. Supplementary Table S4. Characteristics of recipients according to availability of Month 3 UACR, stratified by study cohort. Supplementary Table S5. Supportive longitudinal mixed-effects analyses of UACR and eGFR from Month 1 to Month 3. Supplementary Table S6. Absolute and proportional changes in eGFR and UACR between the silymarin and control groups during the first three months after kidney transplantation. Supplementary Table S7. Lipid profile parameters at Month 1 and Month 3 in silymarin-treated and control kidney transplant recipients. Supplementary Table S8. Sensitivity analyses of LDL cholesterol outcomes adjusted for initiation of lipid-lowering therapy. Supplementary Table S9. Multivariable logistic regression analysis of PTDM at Month 3. Supplementary Table S10. Multivariable logistic regression analysis of prediabetes at Month 3. TREND Statement Checklist.
