# Supplementary material for: Short-Term Silymarin Supplementation After Kidney Transplantation Is Associated with Reduced Early Albuminuria: A Prospective Cohort Study
Source: J Clin Med. 2026 Aug 28;15(17):6694. doi: 10.3390/jcm15176694 (PMC13565761; doi:10.3390/jcm15176694)
Supplement: Supplementary file 1 [file jcm-15-06694-s001.zip › jcm-4497057-supplementary.pdf]

Supplementary material

1. Supplementary Methods

1A. Materials and Methods

1B. Statistical analysis

2. Supplementary Tables

Supplementary Table S1. Laboratory parameters from the day of kidney transplantation to week 2 after transplantation

Supplementary Table S2. Detailed laboratory parameters at month 1 and 2 after kidney transplantation

Supplementary Table S3. Detailed laboratory parameters at month 3 after kidney transplantation

Supplementary Table S4. Characteristics of recipients according to availability of Month 3 UACR, stratified by study cohort.

Supplementary Table S5. Supportive longitudinal mixed-effects analyses of UACR and eGFR from Month 1 to Month 3

Supplementary Table S6. Absolute and proportional changes in eGFR and UACR between the silymarin and control groups during the first three months after kidney transplantation

Supplementary Table S7. Lipid profile parameters at Month 1 and Month 3 in silymarin-treated and control kidney transplant recipients

Supplementary Table S8 Sensitivity analyses of LDL cholesterol outcomes adjusted for initiation of lipid-lowering therapy

Supplementary Table S9. Multivariable logistic regression analysis of PTDM at Month 3

Supplementary Table S10. Multivariable logistic regression analysis of prediabetes at Month 3

3. TREND Statement Checklist

1. Supplementary Methods

1A. Materials and Methods

Study design and population

Silymarin's Advantage on Graft Effectiveness (SAGE) study was designed as a single-center, prospective cohort with historical controls and included patients who underwent kidney transplantation at the Transplant-Nephrology Department of the University Hospital Martin, Slovakia. All consecutive kidney transplant recipients who provided written informed consent during the study period were eligible. Early graft loss and patient death were not exclusion criteria for enrolment. Recipients with unavailable Month 3 urine albumin-to-creatinine ratio (UACR) measurements remained part of the overall study cohort but did not contribute to analyses requiring a Month 3 UACR measurement The only exclusion criteria at enrolment were refusal to participate and known allergy to lactose or any excipient of silymarin tablets.

Patients transplanted between January 2020 and September 2022 who received standard immunosuppressive therapy formed the historical control cohort. From October 2022 until April 2025, consecutive recipients received the same immunosuppressive regimen with the addition of silymarin at a dose of 900 mg per day for 1 month. Follow-up for the present analysis was censored on July 31, 2025, ensuring a minimum follow-up duration of 3 months for all included recipients. Within the final study population, UACR availability varied by follow-up time point. Month 1, Month 2, and Month 3 UACR measurements were available in 76/78, 75/78, and 75/78 recipients in the silymarin cohort and in 57/58, 58/58, and 57/58 recipients in the historical control cohort, respectively. Paired Month 1–Month 3 UACR measurements were available in 75/78 silymarin-exposed recipients and 57/58 controls. Perioperative management and the clinical criteria used for early post-transplant monitoring and intervention were likewise unchanged. All patients were managed at a single transplant center using identical immunosuppressive protocols, biopsy indication criteria, laboratory methods, and follow-up schedules. To minimize potential bias associated with the historical-control design, baseline clinical, demographic, and immunologic characteristics were compared between groups, and multivariable adjustment was applied for key covariates.

The study was investigator-initiated and received Institutional Ethics Committee approval (EK 21/2022) before enrolment of the prospective cohort. The original study concept considered a randomized controlled design; however, practical and regulatory considerations before implementation led to adoption of the prospective cohort design with historical controls that was ultimately conducted. The study was retrospectively registered at

ClinicalTrials.gov (NCT06801886) on January 24, 2025. The registry entry retained elements of the originally planned randomized design and therefore does not fully represent the final implemented methodology.

Donor and recipient characteristics

Detailed demographic, clinical, and immunologic data were collected for all kidney transplant recipients. Recipient characteristics included age at transplantation, sex, body mass index (BMI, kg/m^2), first vs repeat transplantation, aetiology of end-stage kidney disease (ESKD), pre-transplant dialysis modality (hemodialysis or peritoneal dialysis), duration of renal replacement therapy, and time on the transplant waiting list.

Major comorbidities were documented, including:

- disorders of glucose metabolism (type 1 or type 2 diabetes mellitus or prediabetes),
- arterial hypertension,
- liver disease (hepatopathy), defined as structural liver abnormalizes on imaging, persistent liver enzyme elevation, or previously diagnosed chronic liver disease,
- cardiovascular disease, including coronary artery disease, heart failure, clinically significant arrhythmias, or moderate-to-severe valvular disease.

Immunologic risk assessment included human leukocyte antigen (HLA) compatibility (number of HLA-A, HLA-B, and HLA-DR mismatches) and recipient sensitization status, evaluated by actual and maximal historical panel-reactive antibody (PRA) levels. Pre-transplant antibody screening, within 3 months prior to transplantation, was performed using Luminex single-antigen bead (SAB) assays for class I and class II HLA antibodies, including (major histocompatibility complex class I polypeptide–related sequence A) MICA. Donor-specific antibodies (DSA) were identified based on donor HLA typing; positivity, class (I/II), and mean fluorescence intensity (MFI) values were recorded. Donor characteristics included donor age, sex, and donor type (living donor/deceased donor)

Induction immunosuppression

Induction therapy followed a standardized national kidney-transplant immunosuppression protocol applied at our center, using a predefined risk-adapted algorithm integrating immunological and non-immunological factors. The same protocol and risk-stratification criteria were applied throughout both study periods. All recipients received intravenous methylprednisolone 500 mg preoperatively (day 0) and 500 mg on postoperative day 1.

Low immunological risk

Defined by standard-criteria donor status, first transplantation, dialysis duration <3 years, negative Luminex testing, actual and historical PRA < 20%, and cold ischemia time <18 hours. Induction immunosuppression consisted of basiliximab 20 mg on day 0 and day 4.

Moderate risk

This category was subdivided into non-immunologic and immunologic components.

- **Moderate non-immunologic risk:** cold ischemia time >18 hours, presumed delayed graft function, or transplantation from an expanded-criteria donor.
  These recipients received antithymocyte globulin-F (Grafalon) at a cumulative dose of 11 mg/kg (5 mg/kg preoperatively and 3 mg/kg on postoperative days 1 and 2).
- **Moderate immunologic risk:** Luminex positivity without detectable DSA, PRA (actual or historical) 20–50%, or cPRA <95%.
  These recipients received Thymoglobulin at a cumulative dose of 3.5 mg/kg.

High immunological risk

Defined by actual or historical PRA ≥50%, presence of donor-specific antibodies, retransplantation, or a history of a positive crossmatch. Induction consisted of Thymoglobulin at a cumulative dose of 6 mg/kg.

In recipients with DSA between 1000 and 5000 MFI, intravenous immunoglobulin (IVIG) 0.5 g/kg was administered on days 0, 2, and 4. In highly sensitized recipients (DSA >5000 MFI) or in retransplant patients with confirmed DSA, the induction protocol included therapeutic plasma exchange on postoperative days 1, 3, and 5, rituximab 1000 mg on postoperative day 2, and IVIG 2 g/kg on postoperative day 14. Rituximab was therefore reserved for this selected high-immunological-risk subgroup and was not part of routine induction therapy.

Maintenance immunosuppression

Maintenance immunosuppression consisted of tacrolimus, mycophenolic acid, and prednisone. Tacrolimus was initiated at 0.2 mg/kg/day and adjusted to achieve target trough concentrations according to center protocol. Mycophenolic acid was administered at 720 mg twice daily, with dose adjustments based on tolerability and blood counts. Prednisone was administered from postoperative day 2 at 20 mg/day, tapered to 15 mg/day on day 14, and to 10 mg/day on day 30.

Extended criteria donor (ECD)

ECD status was defined according to UNOS criteria (donor age ≥60 years, or age 50–59 years with ≥2 of hypertension, terminal creatinine >132.6 μmol/L, or cerebrovascular cause of death) [11].

Investigation algorithm

Clinical and laboratory assessments followed a predefined study-specific schedule, covering the perioperative and early post-transplant period. At hospital admission for KT, routine biochemical testing was performed, including plasma glucose, urea, and creatinine, and estimated glomerular filtration rate (eGFR) was calculated using the Chronic Kidney Disease Epidemiology Collaboration (CKD-EPI) creatinine equation.

According to the standard protocol of our transplant center, comprehensive biochemical testing was performed on postoperative day 4 (D4), including fasting glucose, urea, creatinine, uric acid, total protein, albumin, a complete lipid profile (total cholesterol [TC], low-density lipoprotein cholesterol [LDL-C], high-density lipoprotein cholesterol [HDL-C], and triglycerides [TAG]), and hepatic enzymes (aspartate aminotransferase [AST], alanine aminotransferase [ALT], alkaline phosphatase [ALP], and gamma-glutamyl transferase [GGT]). Lipid-derived indices were calculated from these values, including LDL/HDL, total cholesterol/HDL (TC/HDL), non-HDL cholesterol, triglyceride/HDL (TAG/HDL), uric acid/creatinine, and uric acid/HDL ratios.

Pre-transplant urinary albumin assessment was not performed because urine production before transplantation was highly heterogeneous. Many recipients were oliguric or anuric due to end-stage kidney disease, whereas others retained variable residual diuresis. Consequently, a standardized pre-transplant urine albumin-to-creatinine ratio could not be obtained or meaningfully compared across participants.

Between postoperative days 4 and 7 (D4–D7), plasma creatinine was measured daily, and the mean creatinine concentration over this interval was used to derive a baseline eGFR (D4–D7), minimizing early postoperative variability. During this interval, delayed graft function (DGF) was documented and defined as the requirement for renal replacement therapy within the first 7 postoperative days [11].

At weeks 1 and 2 post-transplantation, routine biochemical testing (fasting glucose, urea, and creatinine) was repeated, and eGFR was recalculated using the CKD-EPI equation. During the first (M1) and second (M2) post-

transplant months, patients were evaluated at routine outpatient follow-up visits, during which fasting glucose, urea, creatinine, uric acid, total protein, albumin, complete lipid profile, hepatic enzymes, and UACR were obtained.

Urinary albumin assessment. UACR was measured using first-morning spot urine specimens obtained at scheduled Month 1, Month 2, and Month 3 follow-up assessments. Each time-point value represented a single valid clinical measurement rather than the mean of repeated specimens. Urinary albumin and creatinine were analyzed in the same central hospital laboratory using unchanged analytical methodology throughout the study period and in both cohorts. Samples obtained in the presence of clinically apparent urinary tract infection, significant hematuria, active menstruation, or recent urinary tract instrumentation were not considered suitable for scheduled UACR assessment. In such situations, urine testing was deferred until the transient condition had resolved. Scheduled Month 1–3 assessments were performed during ambulatory follow-up, when recipients did not have indwelling urinary catheters or routine urinary instrumentation.

At month 3 (M3), all recipients underwent a scheduled short-term hospital stay for protocol graft biopsy and DSA assessment using Luminex single-antigen bead assays. During this visit, fasting glucose, urea, creatinine, uric acid, total protein, albumin, lipid profile, hepatic enzymes, and UACR were collected, and glycated hemoglobin (HbA1c) was measured. Post-transplant diabetes mellitus (PTDM) was diagnosed according to the 2025 American Diabetes Association criteria. Prediabetes was classified according to the 2024 international consensus. Oral glucose tolerance testing (oGTT) was performed using 75 g of oral glucose dissolved in 200 ml of water, with plasma glucose measured at 1 and 2 hours [12]. These diagnostic criteria were applied retrospectively and uniformly across both study cohorts.

Throughout the study period, data on maintenance immunosuppression were collected, including TAC trough concentration averaged across months 1 (days 0–30), 2 (days 31–60), and 3 (days 61–90). Daily doses of MPA and prednisone were recorded, along with any modifications in immunosuppressive regimen (e.g., conversion between calcineurin inhibitors or mammalian target of rapamycin inhibitors). Recipients were followed on a weekly basis during follow-up. In cases of unexplained graft dysfunction or new-onset proteinuria, for-cause graft biopsy and DSA assessment were performed.

All month-3 protocol biopsy specimens were reviewed for the presence and type of acute rejection and classified as T cell–mediated rejection (TCMR), antibody-mediated rejection (ABMR), or mixed rejection according to the Banff criteria. In addition to protocol biopsies, indication biopsies were performed at any time during follow-up in the presence of graft dysfunction, defined as an increase in serum creatinine ≥20% from baseline, new-onset or worsening proteinuria (urine protein-to-creatinine ratio ≥1.0 g/g or UACR ≥300 mg/g), or detection of de novo donor-specific anti-HLA antibodies. The timing of all biopsy-proven rejection episodes was recorded. Indications for kidney allograft biopsy and histopathological assessment were predefined and remained unchanged throughout the entire study period, including both the control and silymarin-treated cohorts.

Finally, the initiation of lipid-lowering therapy, use of sodium–glucose cotransporter-2 inhibitors (SGLT2i), and occurrence of surgical complications were documented. Surgical complications were defined as any postoperative deviation from the expected course, including urinary leakage, vascular complications, infections, or perinephric fluid collections, in accordance with criteria described by Humar (2005) [12].

1B. Statistical analysis

Statistical analyses were performed using MedCalc v13.1.2 (MedCalc Software Ltd., Ostend, Belgium) and Python (statsmodels and related libraries). Continuous variables are presented as mean ± standard deviation or median (interquartile range), as appropriate, and categorical variables as counts and percentages. Between-group comparisons were conducted using Student's t-test or Mann–Whitney U test for continuous variables and chi-square or Fisher's exact test for categorical variables, as appropriate.

Longitudinal changes in biochemical and metabolic parameters were assessed using nonparametric repeated-measures analyses (Friedman test), with post-hoc pairwise comparisons performed using the Wilcoxon signed-rank test with Bonferroni correction. These methods were selected due to the non-normal distribution of several biomarkers in the early post-transplant period.

Principal renal outcome: UACRUACR exhibited a right-skewed distribution; therefore, log-transformation was applied prior to parametric analyses to improve normality and stabilize variance. Multivariable linear regression was used as a supportive analysis to evaluate UACR at Month 3 with Month 1 UACR and clinically relevant

covariates included in the model. Because Month 1 UACR was measured after the 30-day silymarin exposure period, this model was interpreted as characterizing the subsequent post-treatment UACR trajectory rather than estimating the total effect of silymarin exposure from a pretreatment baseline.

ANCOVA was selected over change-score analysis (ΔUACR) because it provides greater statistical efficiency and is less sensitive to regression-to-the-mean effects. In the present analysis, Month 1 UACR was included as a covariate to account for differences in UACR at the first standardized post-transplant assessment when evaluating the subsequent Month 3 outcome. In addition, multivariable logistic regression was used in an exploratory responder analysis to assess the probability of achieving a ≥25% reduction in UACR between Month 1 and Month 3. This threshold was selected pragmatically to provide a clinically interpretable categorical representation of the subsequent Month 1-to-Month 3 UACR trajectory while retaining adequate event numbers for analysis. It should not be interpreted as an established or validated surrogate threshold in kidney transplant recipients. Previous studies in non-transplant CKD populations have shown that reductions in albuminuria of approximately 30% are associated with improved subsequent kidney outcomes [14]. The primary responder analysis was restricted to recipients with paired UACR measurements at both time points and included 132 recipients: 75/78 recipients in the silymarin cohort and 57/58 controls. Because Month 1 UACR was obtained after the 30-day silymarin exposure period, this analysis was interpreted as characterizing the subsequent post-treatment trajectory rather than change from a pretreatment baseline.

To assess the potential influence of missing Month 3 UACR outcomes, two complementary sensitivity analyses were performed. First, a conservative extreme-case analysis was conducted for the exploratory responder outcome, in which recipients with unavailable Month 3 UACR in the silymarin cohort were classified as nonresponders, whereas recipients with unavailable Month 3 UACR in the control cohort were classified as responders. Second, multiple imputation by chained equations was performed using 50 imputed datasets incorporating available longitudinal UACR measurements and relevant clinical covariates. The adjusted Month 3 continuous UACR model and the adjusted responder model were repeated across the imputed datasets, and pooled estimates and standard errors were obtained using Rubin’s rules. The multiple-imputation analysis was considered a sensitivity analysis under a missing-at-random assumption conditional on the variables included in the imputation model.

Secondary outcomes: eGFR and lipid parameters

For eGFR, multivariable ANCOVA models were used to assess eGFR at M3 with adjustment for eGFR at Month 1 (M1) and relevant clinical covariates. A complementary logistic regression model evaluated the likelihood of achieving a ≥10% increase in eGFR, a threshold selected to exceed expected early post-transplant variability.

Lipid parameters (LDL-C, HDL-C, total cholesterol, non-HDL cholesterol, triglycerides) were analysed using analogous ANCOVA models with adjustment for Month 1 lipid values. This approach was chosen to account for the strong dependence of Month 3 lipid levels on Month 1 values and to avoid biases inherent in change-score analyses

Binary lipid outcomes were defined as ≥15% reduction (LDL-C, total cholesterol, non-HDL-C, triglycerides) or ≥15% increase (HDL-C), reflecting effect sizes considered clinically meaningful in early lipid intervention studies. Logistic regression models were constructed to evaluate these endpoints.

Glycemic outcomes

Multivariable logistic regression models were used to evaluate predictors of post-transplant diabetes mellitus (PTDM) and prediabetic states at M3. Patients with pre-existing diabetes mellitus were excluded from these analyses. Given the relatively low number of events, models were restricted to a limited number of clinically relevant covariates to avoid overfitting.

Covariate selection and model strategy

Covariates were selected based on clinical relevance and biological plausibility, including demographic characteristics (age, sex), transplant-related variables (donor type, induction therapy), immunosuppressive exposure (tacrolimus levels, prednisone dose), and M1 metabolic parameters. Model complexity was limited according to standard principles (approximately one predictor per 10 outcome events), and collinearity was assessed using correlation matrices.

Covariates for multivariable models were selected a priori based on clinical relevance and plausible causal relationships rather than univariable statistical significance. The conceptual framework was based on the assumption that silymarin exposure may influence early albuminuria, lipid parameters, and metabolic outcomes, while these outcomes may also be affected by baseline disease severity, donor characteristics, early graft injury, and immunosuppressive exposure.

For UACR models, Month 1 UACR was included to account for pre-existing albuminuria burden and regression to the mean. Recipient age, ECD donor status, DGF, and basiliximab induction were included as clinically relevant markers of recipient risk, donor quality, early ischemia–reperfusion injury, and immunologic risk. These variables were considered potential confounders because they could influence both treatment allocation or clinical management and early post-transplant albuminuria.

For eGFR models, M1eGFR was included to account for initial graft function. Recipient age, ECD donor status, DGF, and basiliximab induction were selected to capture recipient factors, donor quality, early graft injury, and induction intensity, all of which are established determinants of early graft function recovery.

For lipid models, M1 lipid values were included because month 3 lipid levels are strongly dependent on baseline concentrations. BMI, eGFR, impaired glucose metabolism, prednisone dose, and tacrolimus exposure were selected as biologically plausible determinants of post-transplant lipid metabolism. Initiation of lipid-lowering therapy during follow-up was not included in the primary models because it occurred after M1 assessment and may partly represent a response to lipid levels; therefore, it was evaluated in sensitivity analyses.

For glycemic outcomes, patients with pre-existing diabetes were excluded from PTDM analyses. Models included age, BMI, fasting glucose at M1, prednisone dose, and tacrolimus exposure, reflecting established clinical determinants of post-transplant dysglycemia. Model complexity was intentionally limited because of the low number of glycemic events.

This covariate strategy was intended to adjust for major clinically plausible confounders while avoiding overfitting and adjustment for variables that may lie on the causal pathway between early post-transplant events and outcomes.

Sensitivity analyses

Sensitivity analyses were performed to evaluate the robustness of lipid-related findings with respect to initiation of lipid-lowering therapy during follow-up. Statin initiation between M1 and M3 was included as an additional covariate in both linear (ANCOVA) and logistic regression models. These analyses were considered exploratory.

Handling of missing data

Primary analyses were performed using complete-case data for the relevant outcome. No imputation was used for the primary analyses. Recipients without the measurements required for a specific analysis were excluded only from that analysis and remained included in descriptive and safety analyses. Because Month 3 UACR was unavailable in a small number of recipients, multiple imputation by chained equations was additionally performed as a sensitivity

analysis, as described above. No imputation was applied to the primary analyses of other outcomes. **Sample size estimation**

Sample-size considerations were based on an anticipated clinically meaningful difference in the proportion of recipients achieving a ≥25% reduction in UACR between Month 1 and Month 3. Assuming response rates of 30% in controls and 55% in the silymarin group, 58 recipients per group were estimated to provide 80% power at a two-sided α level of 0.05. This responder threshold was used for sample-size planning and was subsequently analysed as an exploratory categorical outcome. *Ethical approval*

All study procedures were conducted in accordance with the ethical standards of the institutional ethics committee (approval number EK 21/2022), the Declaration of Helsinki, and the Declaration of Istanbul on Organ Trafficking and Transplant Tourism. Written informed consent was obtained from all participants prior to study inclusion. The SAGE study was retrospectively registered at ClinicalTrials.gov (NCT06801886) on January 24, 2025The study was investigator-initiated and received approval from the Institutional Ethics Committee (EK 21/2022) before enrolment of the prospective cohort. The original study concept considered a randomized controlled design. Before implementation of the prospective intervention cohort, practical and regulatory considerations led to adoption of a prospective cohort design with historical controls, which was the design subsequently conducted at the study site. The study was retrospectively registered at ClinicalTrials.gov on January 24, 2025 (NCT06801886) to improve transparency and public accessibility. Because the registry entry retained elements of the originally planned randomized design, it does not fully reflect the final implemented methodology. The present manuscript therefore reports the study according to the design and procedures that were actually implemented and ethically approved. The ≥25% reduction in UACR between Month 1 and Month 3 used for sample-size planning was defined before examination of the final outcome data. During manuscript preparation, the statistical analysis plan was refined to include multivariable adjustment and supportive continuous UACR analyses; these refinements were intended to improve characterization of the post-treatment UACR trajectory and did not alter the responder threshold used for sample-size estimation.

2. Supplementary tables

Supplementary Table S1. Laboratory parameters from the day of kidney transplantation to week 2 after transplantation

Parameter	Silymarin Group (n = 78)	Control Group (n = 58)	P value
**Day of kidney transplantation**			
Glucose (mmol/L))	6.78 ± 3.83, median 5.1	6.7 ± 5.8, median 5.3	0.3522
Urea (mmol/L)	20.8 ± 17.3, median 14.75	19.7 ± 14.7, median 28.4	0.2985
Creatinine (µmol/L))	669 ± 247, median 631	638 ± 219, median 596	0.5594
eGFR (mL/min/1.73 m²))	8.3 ± 4.4, median 7	9.6 ± 5.5, median 9	0.0643
**Day 4 after kidney transplantation**			
Glucose (mmol/L))	5.98 ± 2.37, median 5.1	6.64 ± 2.99, median 5.95	0.0616
Urea (mmol/L))	14.4 ± 7, median 12.9	14.8 ± 7.5, median 13.6	0.7686
Creatinine (µmol/L)	319 ± 233, median 211	268 ± 179, median 206	0.3320
eGFR (mL/min/1.73 m²)	27.7 ± 17, median 25.5	36 ± 26, median 31.5	0.0912
Uric acid (µmol/L))	251.6 ± 78.3, median 237	313 ± 109.3, median 307.5	**0.0005**
Total protein (g/L))	51.6 ± 5.3, median 51.7	51.2 ± 4.8, median 51.3	0.6677
Albumin (g/L))	31 ± 3.7, median 31.2	30.7 ± 2.9, median 30.5	0.6550
Total cholesterol (mmol/L))	3.6 ± 0.9, median 3.5	3.9 ± 1, median 3.8	0.1077
Triglycerides (mmol/L))	2.6 ± 1.3, median 2.3	2.7 ± 1, median 2.6	0.1254
High-density lipoprotein cholesterol (HDL, mmol/L)	1 ± 0.5, median 0.8	1.2 ± 0.4, median 1.1	**< 0.0001**
Low-density lipoprotein cholesterol (LDL, mmol/L))	1.9 ± 0.7, median 1.8	2.9 ± 1.4, median 2.5	**< 0.0001**
Non-HDL cholesterol (mmol/L))	2.6 ± 1.7, median 2.7	2.7 ± 1, median 2.5	0.8768
Ratio LDL/HDL	2.45 ± 1.2, median 2.31	2.5 ± 0.9, median 2.6	0.3422
Ratio total choles-terol/HDL	4.57 ± 1.91, median 4.23	3.6 ± 1.31, median 3.45	**0.0017**
Ratio triglycerides/HDL	3.45 ± 2.62, median 2.8	2.7 ± 1.6, median 2.3	0.0652
Ratio uric acid/HDL	313.6 ± 160, median 292.1	302 ± 173.2, median 254.5	0.3468
Ratio uric acid/creatinine	1.12 ± 0.68, median 0.95	1.56 ± 0.88, median 1.39	**0.0030**
Gamma-glutamyl transfer-ase (GGT, µkat/L))	0.75 ± 0.73, median 0.56	0.98 ± 1.48, median 0.53	0.6664
Aspartate aminotransfer-ase (AST, µkat/L))	0.48 ± 0.23, median 0.41	0.47 ± 0.2, median 0.42	0.7294

Alanine aminotransferase (ALT, µkat/L))	0.53 ± 0.38, median 0.4	0.49 ± 0.32, median 0.37	0.6468
Alkaline phosphatase (ALP, µkat/L))	1 ± 0.4, median 1	1.02 ± 0.45, median 0.91	0.5034
**Week one after kidney transplantation**			
Glucose (mmol/L))	6.7 ± 5.4, median 5.3	6.18 ± 2.57, median 5.5	0.6038
Urea (mmol/L))	14.3 ± 9.7, median 11.2	14.1 ± 9.35, median 11.2	0.9035
Creatinine (µmol/L)	252 ± 207, median 172	220 ± 158, median 171	0.6245
eGFR (mL/min/1.73 m^2)	37 ± 20.3, median 35.5	46 ± 31.4, median 36	0.1989
**Week 2 after kidney transplantation**			
Glucose (mmol/L))	7.2 ± 4.2, median 5.8	7.79 ± 5.6, median 5.8	0.5682
Urea (mmol/L))	12.1 ± 6.1, median 9.9	14.6 ± 15.4, median 15.4	0.6582
Creatinine (µmol/L),)	206 ± 127, median 174	188 ± 113, median 156	0.1562
eGFR (mL/min/1.73 m^2))	37.2 ± 17, median 37	46 ± 24.2, median 46	**0.0266**

ALP, alkaline phosphatase; ALT, alanine aminotransferase; AST, aspartate aminotransferase; eGFR, estimated glomerular filtration rate; GGT, gamma-glutamyl transferase; HDL, high-density lipoprotein cholesterol; LDL, low-density lipoprotein cholesterol; SD, standard deviation.

Laboratory parameters measured from the day of kidney transplantation through week 2 after transplantation in silymarin-treated and control recipients. Biochemical, renal function, lipid, and hepatic parameters are presented at predefined early post-transplant time points. Continuous variables are shown as mean ± standard deviation with median values. Between-group comparisons were performed using appropriate statistical tests

Supplementary Table S2. Detailed laboratory parameters at month 1 and 2 after kidney transplantation

Parameter	Month 1 after kidney transplantation			Month 2 after kidney transplantation		
	Silymarin Group (n = 78)	Control Group (n = 58)	P value	Silymarin Group (n = 78)	Control Group (n = 58)	P value
Glucose (mmol/L)	7.32 ± 3.88, median 5.55	6.94 ± 2.74, median 5.6	0.8222	6.03 ± 2.12, median 5.2	5.82 ± 2.2, median 5.3	0.7748
Urea (mmol/L))	10.2 ± 5.6, median 9	11.1 ± 5.9, median 9.4	0.3631	9.4 ± 3.6, median 8.4	12.3 ± 5.6, median 8.3	0.7802
Creatinine (μmol/L))	180 ± 109, median 154	175 ± 96.4, median 137	0.5817	161 ± 62.6, median 143	171 ± 105, median 138	0.5076
eGFR (mL/min/1.73 m²))	43.6 ± 17.2, median 42	49.1 ± 24.1, median 47	0.1266	46 ± 16, median 45	52.1 ± 26.1, median 49.5	0.2326
Uric acid (μmol/L))	333 ± 81, median 319	359.3 ± 111, median 352.5	**0.0379**	364 ± 83.7, median 343.5	369 ± 96.8, median 361	0.7790
Total protein (g/L))	64.5 ± 5.6, median 64.7	75.3 ± 8.2, median 65	0.8769	65.6 ± 9.3, median 67.3	65.9 ± 7.3, median 66.4	0.8223
Albumin (g/L))	44.8 ± 5.1, median 39.4	45.3 ± 4.5, median 40	0.5548	41 ± 5.7, median 41.9	40.6 ± 5.2, median 41	0.2998
Total cholesterol (mmol/L)	4.12 ± 1.64, median 3.71	6.16 ± 1.44, median 6.3	**< 0.0001**	4.77 ± 0.96, median 4.7	5.85 ± 1.48, median 5.75	**<0.0001**
Triglycerides (mmol/L)	2.1 ± 1, median 1.95	2.69 ± 1.23, median 2.45	**0.0020**	1.89 ± 0.82, median 1.7	2.39 ± 1.03, median 2.1	**0.0013**
High-density lipoprotein cholesterol (HDL))	1.46 ± 0.3, median 1.26	1.57 ± 0.48, median 1.5	**0.0074**	1.28 ± 0.4, median 1.25	1.49 ± 0.48, median 1.45	**0.0093**
Low-density lipoprotein cholesterol (LDL))	3.04 ± 1.8, median 2.8	3.84 ± 1.38, median 3.8	**< 0.0001**	2.67 ± 0.8, median 2.6	3.66 ± 1.24, median 3.6	**<0.0001**
Non-HDL cholesterol (mmol/L))	3.58 ± 1.8, median 3.6	4.6 ± 1.29, median 4.6	**0.0001**	3.49 ± 0.81, median 3.46	4.36 ± 1.3, median 4.25	**<0.0001**
Ratio LDL/HDL	2.56 ± 2.28, median 2.11	2.64 ± 0.96, median 2.63	0.0625	2.25 ± 0.95, median 2.08	2.6 ± 0.97, median 2.4	**0.0144**
Ratio total cholesterol/HDL	4.12 ± 1.64, median 3.71	4.15 ± 1.16, median 4.04	0.4755	3.97 ± 1.16, median 3.82	4.19 ± 1.31, median 4.06	0.3360
Ratio triglycerides/HDL	1.89 ± 1.6, median 1.41	1.91 ± 1.12, median 1.56	0.3395	1.69 ± 1.19, median 1.42	1.83 ± 1.05, median 1.54	0.3493
Ratio uric acid/HDL	292 ± 114, median 244	255.3 ± 116.4, median 246	0.5938	322 ± 159.2, median 298.3	292 ± 195, median 252	0.0538

Ratio uric acid/creatinine	2.1 ± 0.6, median 2.04	2.31 ± 0.82, median 2.22	0.0593	2.45 ± 0.73, median 2.4	2.49 ± 0.78, median 2.54	0.7356
Gamma-glutamyl transferase (GGT, µkat/L))	0.95 ± 0.86, median 0.63	1.42 ± 1.35, median 0.93	**0.0040**	0.78 ± 1.34, median 0.49	0.97 ± 1.09, median 0.64	**0.0159**
Aspartate aminotransferase (AST, µkat/L))	0.4 ± 0.26, median 0.33	0.42 ± 0.17, median 0.36	0.1020	0.44 ± 0.21, median 0.38	0.42 ± 0.15, median 0.4	0.9367
Alanine aminotransferase (ALT, µkat/L)	0.7 ± 0.71, median 0.45	0.71 ± 0.57, median 0.36	0.4099	0.55 ± 0.53, median 0.38	0.53 ± 0.41, median 0.43	0.4074
Alkaline phosphatase (ALP, µkat/L)	1.57 ± 0.68, median 1.42	1.72 ± 0.86, median 1.61	0.3331	1.51 ± 1.28, median 1.27	1.5 ± 0.6, median 1.46	0.2883
Urine albumin-to-creatinine ratio (UACR, mg/mmol))	12.86 ± 13.64, median 8.1	26.63 ± 25.45, median 21.2	**< 0.0001**	7.01 ± 8.68, median 3.8	26.19 ± 50.51, median 13.1	**<0.0001**
Calcineurin inhibitor (CNI) therapy – tacrolimus	78 (100%)	58 (100%)	1.0000	77 (98.7%)	58 (100%)	0.0737
- Tacrolimus trough level (ng/mL))	14.1 ± 3.2, median 13.5	15.5 ± 3.5, median 15.1	0.1067	13.6 ± 3.29, median 13	13.4 ± 3.8, median 12.8	0.4364
Mycophenolic acid (MPA) (%)	76 (97.4%)	57 (98.3%)	0.7424	75 (96.2%)	55 (94.8%)	0.6999
- MPA daily dose (mg/day))	1028 ± 173.4, median 1080	1213 ± 316, median 1440	**< 0.0001**	935.5 ± 259, median 1080	1021 ± 257, median 1080	**0.0333**
Corticosteroid use (%)	78 (100%)	58 (100%)	1.0000	77 (98.7%)	56 (96.6%)	0.5752
- prednisone daily dose (mg/day))	14.4 ± 1.8, median 15	14.6 ± 1.42, median 15	0.5893	10.1 ± 0.57, median 10	9.9 ± 0.66, median 10	0.1547

ALP, alkaline phosphatase; ALT, alanine aminotransferase; AST, aspartate aminotransferase; CNI, calcineurin inhibitor; eGFR, estimated glomerular filtration rate; GGT, gamma-glutamyl transferase; HDL, high-density lipoprotein cholesterol; LDL, low-density lipoprotein cholesterol; MPA, mycophenolic acid; SD, standard deviation; UACR, urine albumin-to-creatinine ratio.

Detailed laboratory, metabolic, and immunosuppressive parameters assessed at month 1 and month 2 after kidney transplantation in silymarin-treated and control recipients. Renal function, glycaemic, lipid, hepatic, and immunosuppression-related variables are presented at predefined follow-up time points. Continuous variables are shown as mean ± standard deviation with median values, and categorical variables as percentages. Between-group comparisons were performed using appropriate statistical tests.

Supplementary Table S3. Detailed laboratory parameters at month 3 after kidney transplantation

Parameter	Silymarin Group (n = 78)	Control Group (n = 58)	P value
Glucose (mmol/L))	6.01 ± 1.92, median 5.2	5.75 ± 1.87, median 5.3	0.7198
Urea (mmol/L))	8.6 ± 3.9, median 7.6	9.2 ± 4.7, median 7.7	0.7775
Creatinine (µmol/L))	144.5 ± 58, median 134	149 ± 72, median 122	0.5186
eGFR (mL/min/1.73 m²))	51.3 ± 15.4, median 49	57.4 ± 27.2, median 55	0.1007
Uric acid (µmol/L))	375.2 ± 86.5, median 356.5	368.2 ± 81.5, median 351	0.7572
Total protein (g/L))	66.3 ± 4.9, median 66.5	64.6 ± 5.5, median 64.5	0.0796
Albumin (g/L))	40.5 ± 4.9, median 41	40.5 ± 3.9, median 40.6	0.5893
Total cholesterol (mmol/L))	4.73 ± 0.93, median 4.8	5.35 ± 1.13, median 5.3	**0.0008**
Triglycerides (mmol/L))	1.91 ± 1, median 1.7	2.2 ± 1.1, median 1.9	0.0937
High-density lipoprotein cholesterol (mmol/L))	1.29 ± 0.4, median 1.2	1.42 ± 0.48, median 1.3	0.2144
Low-density lipoprotein cholesterol (mmol/L))	2.74 ± 0.76, median 2.7	3.26 ± 0.99, median 3.3	**0.0008**
Non-HDL cholesterol (mmol/L))	3.45 ± 0.8, median 3.42	3.93 ± 1.05, median 3.8	**0.0032**
Ratio LDL/HDL	2.32 ± 0.94, median 2.17	2.49 ± 0.95, median 2.54	0.2873
Ratio total cholesterol/HDL	3.96 ± 1.29, median 3.58	3.33 ± 1.42, median 3.24	**0.0063**
Ratio triglycerides/HDL	1.73 ± 1.38, median 1.2	1.77 ± 1.2, median 1.44	0.6328
Ratio uric acid/HDL	334 ± 205.3, median 289.2	294.7 ± 132.5, median 275	0.3463
Ratio uric acid/creatinine	2.72 ± 0.64, median 2.57	2.75 ± 0.81, median 2.67	0.8198
Gamma-glutamyl transferase (GGT, µkat/L))	0.81 ± 1.07, median 0.51	0.91 ± 1, median 0.5	0.2885
Aspartate aminotransferase (AST, µkat/L))	0.55 ± 0.27, median 0.47	0.48 ± 0.24, median 0.44	0.1165
Alanine aminotransferase (ALT, µkat/L))	0.72 ± 0.65, median 0.48	0.66 ± 0.61, median 0.5	0.7339
Alkaline phosphatase (ALP, µkat/L)	1.4 ± 0.63, median 1.3	1.41 ± 0.64, median 1.35	0.9049
Urine albumin-to-creatinine ratio (UACR, mg/mmol))	7.04 ± 10.9, median 3.7	23.8 ± 44.7, median 13.8	**<0.0001**
C-peptide - fasting (ng/mL))	4.6 ± 2.4, median 3.6	4.27 ± 2.24, median 3.52	0.5361
C-peptide - 2 h postprandial (ng/mL))	13.8 ± 12.1, median 12.2	11.4 ± 5.94, median 12.3	0.7418

Insulin - fasting (mU/L))	8.3 ± 6.8, median 6.3	10.1 ± 9.1, median 7.9	0.0582
Insulin - 2 h postprandial (mU/L))	27.1 ± 17.8, median 26.6	45.6 ± 43.6, median 29.3	0.1744
Glycated hemoglobin (HbA1c, %)	4.2 ± 1.1, median 4	4.62 ± 1.57, median 4.3	0.1656
Calcineurin inhibitor (CNI) therapy – tacrolimus	76 (97.4%)	54 (93.1%)	0.4012
Tacrolimus trough level (ng/mL))	10.7 ± 2.35, median 9.7	10.6 ± 2.6, median 10.6	0.3176
Mycophenolic acid (MPA)	68 (87.2%)	53 (91.4%)	0.0701
- MPA daily dose (mg/day))	842 ± 272, median 1080	871 ± 306, median 1080	0.5359
Corticosteroid use	76 (97.4%)	56 (96.6%)	0.7636
- prednisone daily dose (mg/day))	10.4 ± 1.5, median 10	9.9 ± 0.74, median 10	0.7636
History of ABMR	0 (0%)	3 (5.2%)	0.0890
History of TCMR	3 (3.8%)	5 (8.6%)	0.3300
- time from KTx to TCMR (months))	2.83 ± 0.41, median 3	2.43 ± 0.98, median 3	0.4978
- time from KTx to ABMR (months),	-	2.33 ± 1.15, median 3	-
Donor-specific antibody positive	4 (5.1%)	6 (10.3%)	0.3242
- Class I (Mean fluorescence intensity – MFI))	2445 ± 1841, median 1652	2693 ± 3000, median 1780	1.0000
- Class II (Mean Fluorescence Intensity – MFI))	4380 ± 4780, median 2305	2700 ± 2700, median 283	0.8000
MICA antibodies, positive	1 (1.3%)	1 (1.7%)	0.8329
Post-transplant diabetes mellitus development	9 (11.5%)	8 (13.8%)	0.4349
Impaired Glucose Tolerance development	15 (19.2%)	5 (8.6%)	0.1407
Impaired Fasting Glucose development	0 (0%)	1 (1.7%)	0.2462
Lipid-lowering therapy	8 (10.3%)	13 (22.4%)	0.059
- time from KTx to initiation of lipid-lowering therapy (months))	2 ± 0.8, median 2	1.75 ± 0.75, median 2	0.4330

ABMR, antibody-mediated rejection; ALP, alkaline phosphatase; ALT, alanine aminotransferase; AST, aspartate aminotransferase; CNI, calcineurin inhibitor; DSA, donor-specific antibodies; eGFR, estimated glomerular filtration rate; GGT, gamma-glutamyl transferase; HDL, high-density lipoprotein cholesterol; LDL, low-density lipoprotein cholesterol; MFI, mean fluorescence intensity; MICA, major histocompatibility complex class I polypeptide–related sequence A; MPA, mycophenolic acid; SD, standard deviation; TCMR, T cell–mediated rejection; UACR, urine albumin-to-creatinine ratio.

Detailed laboratory, metabolic, immunologic, and immunosuppressive parameters assessed at month 3 after kidney transplantation in silymarin-treated and control recipients. Renal function, albuminuria, lipid profile, glycaemic indices, liver enzymes, immunologic outcomes, and maintenance immunosuppression exposure are summarized. Continuous variables are presented as mean ± standard deviation with median values, and categorical variables as percentages with absolute counts. Between-group comparisons were performed using appropriate statistical tests.

Supplementary Table S4. Characteristics of recipients according to availability of Month 3 UACR, stratified by study cohort.

Parameter	Silymarin, M3 UACR available (n=75)	Silymarin, M3 UACR unavailable (n=3)	Control, M3 UACR available (n=57)	Control, M3 UACR unavailable (n=1)
Age, years	48.75 ± 13.49; median 50 (39–58)	63.33 ± 6.81; median 61 (59.5–66)	43.40 ± 15.66; median 44 (31–57)	76
BMI, kg/m²	26.12 ± 4.45; median 26.2 (22.8–28.6)	26.20 ± 1.42; median 26.7 (25.7–27.0)	26.56 ± 5.31; median 26.9 (22.9–30.5)	23.2
eGFR at Month 1, mL/min/1.73 m²	43.60 ± 17.30; median 42 (32.5–55.0)	42.0 (n=1)	49.75 ± 23.78; median 47 (31–64)	10.0
UACR at Month 1, mg/mmol	12.97 ± 13.69; median 8.2 (4.1–15.8)	4.3 (n=1)	27.04 ± 25.25; median 21.7 (10.7–31.2)	Not available
UACR at Month 2, mg/mmol	7.01 ± 8.68; median 3.8 (1.8–8.4)	Not available	25.28 ± 50.49; median 13.0 (6.9–26.0)	77.7
Male sex, n (%)	46/75 (61.3%)	2/3 (66.7%)	36/57 (63.2%)	1/1 (100%)
ECD donor, n (%)	21/75 (28.0%)	1/3 (33.3%)	13/57 (22.8%)	1/1 (100%)
Delayed graft function, n (%)	7/75 (9.3%)	1/3 (33.3%)	3/57 (5.3%)	1/1 (100%)
Basiliximab induction, n (%)	22/75 (29.3%)	0/3 (0%)	25/57 (43.9%)	0/1 (0%)

Data are presented descriptively because Month 3 UACR was unavailable in only four recipients (three in the silymarin cohort and one in the control cohort); therefore, no inferential comparisons were performed. Continuous variables are presented as mean ± standard deviation and median (interquartile range), where applicable, and categorical variables as n/N (%). For variables with additional missing measurements, the available denominator is indicated in the corresponding cell. BMI, body mass index; ECD, expanded criteria donor; eGFR, estimated glomerular filtration rate; DGF, delayed graft function; M3, Month 3; UACR, urine albumin-to-creatinine ratio.

Supplementary Table S5. Supportive longitudinal mixed-effects analyses of UACR and eGFR from Month 1 to Month 3

Outcome / term	Estimate	95% CI	P value
**Log-transformed UACR**			
Silymarin cohort at Month 1	−0.899	−1.266 to −0.533	<0.001
Month 2 vs Month 1, control cohort	−0.387	−0.624 to −0.150	0.001
Month 3 vs Month 1, control cohort	−0.470	−0.707 to −0.232	<0.001
Silymarin × Month 2 interaction	−0.322	−0.637 to −0.007	0.045
Silymarin × Month 3 interaction	−0.339	−0.654 to −0.024	0.035
Age, per year	−0.001	−0.012 to 0.011	0.920
ECD donor	0.232	−0.162 to 0.627	0.248
Delayed graft function	0.570	−0.011 to 1.150	0.054
Basiliximab induction	−0.042	−0.390 to 0.307	0.815
**Overall cohort × time interaction**	LR χ^2 = 5.59, df = 2	—	**0.061**
**Adjusted geometric mean ratio, silymarin vs control**			
Month 1	0.407	0.282 to 0.587	<0.001
Month 2	0.295	0.205 to 0.425	<0.001
Month 3	0.290	0.201 to 0.418	<0.001
**eGFR**			
Silymarin cohort at Month 1	−2.809	−9.227 to 3.609	0.391
Month 2 vs Month 1, control cohort	3.017	−0.235 to 6.270	0.069
Month 3 vs Month 1, control cohort	7.658	4.385 to 10.931	<0.001
Silymarin × Month 2 interaction	−0.516	−4.846 to 3.814	0.815
Silymarin × Month 3 interaction	−0.037	−4.382 to 4.309	0.987
Age, per year	−0.282	−0.500 to −0.065	0.011
ECD donor	−9.134	−16.485 to −1.783	0.015
Delayed graft function	−18.144	−28.893 to −7.395	0.001
Basiliximab induction	2.748	−3.754 to 9.249	0.407
**Overall cohort × time interaction**	LR χ^2 = 0.07, df = 2	—	**0.967**
**Adjusted eGFR difference, silymarin vs control, mL/min/1.73 m²**			
Month 1	−2.81	−9.23 to 3.61	0.391
Month 2	−3.32	−9.75 to 3.10	0.310
Month 3	−2.85	−9.28 to 3.59	0.386

UACR, urine albumin-to-creatinine ratio; eGFR, estimated glomerular filtration rate; ECD, expanded criteria donor; CI, confidence interval; GMR, geometric mean ratio; LR, likelihood-ratio. Supportive longitudinal analyses were performed using linear mixed-effects models with a patient-specific random intercept. Time was modeled as a categorical variable with Month 1 as the reference time point. Models included study cohort, time, cohort-by-time interaction, recipient age, ECD status, delayed graft function, and basiliximab induction. UACR was log-transformed because of its right-skewed distribution. For the UACR model, regression coefficients are expressed on the log scale; model-based between-group contrasts were exponentiated and are additionally presented as adjusted geometric mean ratios. For eGFR, coefficients and contrasts are expressed in mL/min/1.73 m^2. The overall cohort-by-time interaction was evaluated using a likelihood-ratio test comparing models with and without the interaction terms. The UACR model included 134 recipients contributing 398 observations, and the eGFR model included 134 recipients contributing 399 observations. Because Month 1 UACR represented the first standardized post-treatment assessment, these longitudinal analyses characterize the subsequent post-treatment trajectory and should not be interpreted as estimating the total causal effect of silymarin exposure.

Supplementary Table S6. Absolute and proportional changes in eGFR and UACR between the silymarin and control groups during the first three months after kidney transplantation

Parameter	Silymarin	Control	P value
Absolute change in eGFR			
Δ eGFR D4–D7 to M1 (mL/min/1.73 m²)	11.8 (1.5–21.5)	6.5 (−2.2–21.6)	0.2923
Δ eGFR D4–D7 to M2 (mL/min/1.73 m²)	14.5 (4.5–24.5)	11.5 (−0.9–23.1)	0.4153
Δ eGFR D4–D7 to M3 (mL/min/1.73 m²)	18.5 (8.5–31.2)	14.5 (2.5–24.0)	0.1452
Δ eGFR M1 to M2 (mL/min/1.73 m²)	3.0 (−4.0–8.0)	4.0 (−2.0–11.0)	0.5611
Δ eGFR M1 to M3 (mL/min/1.73 m²)	9.0 (1.5–13.5)	7.0 (0.0–18.0)	0.7216
Relative change in eGFR			
Δ eGFR D4–D7 to M1 (%)	39.0 (3.7–96.2)	22.3 (−5.7–83.4)	0.1342
Δ eGFR D4–D7 to M2 (%)	44.0 (10.8–154.4)	35.5 (−1.8–85.1)	0.1596
Δ eGFR D4–D7 to M3 (%)	63.1 (21.6–170.8)	42.9 (4.1–100.0)	0.1119
Δ eGFR M1 to M2 (%)	7.0 (−6.5–19.5)	9.0 (−10.4–26.4)	0.9187
Δ eGFR M1 to M3 (%)	20.9 (4.1–41.4)	13.0 (0.0–43.2)	0.4135
Absolute change in UACR			
Δ UACR M1 to M2 (mg/mmol)	−3.7 (−8.1 to −0.4)	−3.4 (−13.9 to −1.2)	0.5488
Δ UACR M1 to M3 (mg/mmol)	−4.1 (−10.2 to 0.2)	−3.7 (−13.1 to 1.0)	0.9817
Relative change in UACR			
Δ UACR M1 to M2 (%)	−54.2 (−71.9 to −2.0)	−31.5 (−55.7 to −7.5)	**0.0391**
Δ UACR M1 to M3 (%)	−60.0 (−80.4 to 5.0)	−21.5 (−60.2 to 3.9)	**0.0396**

D4–D7, postoperative days 4–7; M1, month 1; M2, month 2; M3, month 3; eGFR, estimated glomerular filtration rate; UACR, urinary albumin-to-creatinine ratio.

Between-group comparisons were performed using the Mann–Whitney U test. Absolute changes are expressed in mL/min/1.73 m² for eGFR and mg/mmol for UACR, whereas proportional changes are expressed as percentages relative to the earlier measurement in each specified interval. For UACR, Month 1 represents the first standardized post-treatment assessment. Accordingly, Month 1-

to-Month 2 and Month 1-to-Month 3 changes describe the subsequent post-treatment trajectory and should not be interpreted as changes from a pretreatment baseline. Statistically significant P values ($P < 0.05$) are shown in bold

Supplementary Table S7. Lipid profile parameters at Month 1 and Month 3 in silymarin-treated and control kidney transplant recipients

Parameter (mmol/L)	Silymarin (M1)	Control (M1)	p value	Silymarin (M3)	Control (M3)	P value
**Total cholesterol**	4.12 ± 1.64	6.16 ± 1.44	**<0.0001**	4.73 ± 0.93	5.35 ± 1.13	**0.0008**
**LDL-C**	3.04 ± 1.80	3.84 ± 1.38	**<0.0001**	2.74 ± 0.76	3.26 ± 0.99	**0.0008**
**HDL-C**	1.46 ± 1.30	1.57 ± 0.48	**0.0074**	1.29 ± 0.40	1.42 ± 0.48	0.2144
**Triglycerides**	2.10 ± 1.00	2.69 ± 1.23	**0.0020**	1.91 ± 1.0	2.20 ± 1.1	0.0937
**Non-HDL-C**	3.58 ± 1.80	4.60 ± 1.29	**0.0001**	3.45 ± 0.80	3.93 ± 1.05	**0.0032**

HDL-C, high-density lipoprotein cholesterol ; LDL-C, low-density lipoprotein cholesterol; M1, month 1; M2, month 2; M3, month 3; non-HDL-C, non-high-density lipoprotein cholesterol

Lipid profile parameters at 1 and 3 months after kidney transplantation in silymarin-treated and control recipients. Total cholesterol, LDL-cholesterol, HDL-cholesterol, triglycerides, and non-HDL-cholesterol are presented as the mean ± standard deviation. Between-group comparisons were performed at each time point using the appropriate statistical tests.

Supplementary Table S8. Sensitivity analyses of LDL cholesterol outcomes adjusted for initiation of lipid-lowering therapy

**Variable**	**≥15% reduction in LDL cholesterol**			**LDL cholesterol at Month 3**		
	OR	95% CI	P value	Beta	95% CI	P value
Silymarin treatment	1.10	0.42–2.89	0.846	-0.30	-0.60–0.01	0.055
LDL cholesterol at Month 1	**3.24**	**1.88–5.59**	**<0.001**	**0.15**	**0.06–0.25**	**0.001**
Body mass index	0.95	0.86–1.04	0.269	0.02	-0.01–0.05	0.162
eGFR at Month 1	0.99	0.97–1.02	0.563	**0.01**	**0.00–0.02**	**0.028**
Impaired glucose metabolism	1.19	0.38–3.67	0.768	-0.15	-0.51–0.20	0.396
Prednisone dose at Month 1	**0.67**	**0.49–0.92**	**0.014**	**0.12**	**0.03–0.22**	**0.012**
Tacrolimus (mean level)	1.07	0.85–1.34	0.587	-0.02	-0.09–0.05	0.641
Statin initiation	2.33	0.64–8.44	0.199	-0.37	-0.79–0.05	0.083

CI, confidence interval; eGFR, estimated glomerular filtration rate; M1, Month 1; M3, Month 3; LDL, low-density lipoprotein. Multivariable logistic and linear regression models were constructed to assess LDL cholesterol outcomes, including ≥15% reduction between M1 and M3 and LDL cholesterol at M3, respectively. All models were adjusted for LDL cholesterol at M1, body mass index, estimated glomerular filtration rate at M1, impaired glucose metabolism, prednisone dose at M1, tacrolimus exposure, and initiation of lipid-lowering therapy during follow-up.

Odds ratios (OR) and beta coefficients represent adjusted associations with the respective outcomes.

Statistically significant P values ($P < 0.05$) are shown in bold.

Supplementary Table S9. Multivariable logistic regression analysis of PTDM at Month 3

Variable	OR	95% CI	P value
Silymarin treatment	0.67	0.19-2.39	0.540
Age	1.02	0.97-1.07	0.386
Body mass index	1.08	0.95-1.23	0.263
Fasting glucose at Month 1	1.02	0.94-1.10	0.680
Prednisone dose at Month 1	0.92	0.57-1.47	0.719
Tacrolimus (mean level)	0.67	0.67–1.17	0.390

OR, odds ratio; CI, confidence interval; M1, Month 1; M3, Month 3; PTDM, post-transplant diabetes mellitus.

Multivariable logistic regression analysis was performed to evaluate factors associated with PTDM at M3. Patients with pre-existing diabetes mellitus were excluded. The model was adjusted for age, body mass index, fasting glucose at M1, prednisone dose at M1, and tacrolimus exposure.

Supplementary Table S10. Multivariable logistic regression analysis of prediabetes at Month 3

Variable	OR	95% CI	P value
Silymarin treatment	2.47	0.70–8.72	0.160
Age	1.03	0.99–1.08	0.162
Body mass index	1.05	0.93–1.18	0.407
Fasting glucose at Month 1	0.92	0.66–1.29	0.646
Prednisone dose at Month 1	0.92	0.63–1.34	0.671
Tacrolimus (mean level)	0.92	0.72–1.17	0.481

OR, odds ratio; CI, confidence interval; M1, Month 1; M3, Month 3.

Multivariable logistic regression analysis was performed to evaluate factors associated with prediabetes at M3. Patients with pre-existing diabetes mellitus were excluded. The model was adjusted for age, body mass index, fasting glucose at M1, prednisone dose at M1, and tacrolimus exposure.

TREND Statement Checklist

Completed checklist for the Transparent Reporting of Evaluations with Nonrandomized Designs (TREND) statement.

Paper Section/Topic	Item No.	Descriptor	Reported? ✓	Pg #
**TITLE and ABSTRACT**				
Title and Abstract	1	• Information on how units were allocated to interventions	✓	1
		• Structured abstract recommended	✓	1
		• Information on target population or study sample	✓	1
**INTRODUCTION**				
Background	2	• Scientific background and explanation of rationale	✓	2-3
		• Theories used in designing behavioral interventions	N/A*	
**METHODS**				
Participants	3	✓Eligibility criteria for participants, including criteria at different levels in recruitment/sampling plan (e.g., cities, clinics, subjects)	✓	3; Supp. 33
		✓Method of recruitment (e.g., referral, self-selection), including the sampling method if a systematic sampling plan was implemented	✓	3; Supp. 33
		✓Recruitment setting	✓	3
		✓Settings and locations where the data were collected	✓	3–5; Supp. 33-39
Interventions	4	• Details of the interventions intended for each study condition and how and when they were actually administered, specifically including:	✓	3–6; Supp. 33-36
		o Content: what was given?	✓	6
		o Delivery method: how was the content given?	✓	6
		o Unit of delivery: how were subjects grouped during delivery?	✓	3, 6
		o Deliverer: who delivered the intervention?	✓	6
		o Setting: where was the intervention delivered?	✓	3-6

		o Exposure quantity and duration: how many sessions or episodes or events were intended to be delivered? How long were they intended to last?	✓	6
		o Time span: how long was it intended to take to deliver the intervention to each unit?	✓	6
		o Activities to increase compliance or adherence (e.g., incentives)	✓	6
Objectives	5	• Specific objectives and hypotheses	✓	3
Outcomes	6	• Clearly defined primary and secondary outcome measures	✓	5-6
		• Methods used to collect data and any methods used to enhance the quality of measurements	✓	5; Supp. 36-39
		• Information on validated instruments such as psychometric and biometric properties	N/A**	
Sample size	7	• How sample size was determined and, when applicable, explanation of any interim analyses and stopping rules	✓	Supp. 43
Assignment method	8	• Unit of assignment (the unit being assigned to study condition, e.g., individual, group, community)	✓	3
		• Method used to assign units to study conditions, including details of any restriction (e.g., blocking, stratification, minimization)	✓	3-4; Supp. 33-34
		• Inclusion of aspects employed to help minimize potential bias induced due to non-randomization (e.g., matching)	✓	3-4; Supp. 33-34, 41–43
Blinding (masking)	9	• Whether or not participants, those administering the interventions, and those assessing the outcomes were blinded to study condition assignment; if so, statement regarding how the blinding was accomplished and how it was assessed	△***	
Unit of Analysis	10	• Description of the smallest unit that is being analysed to assess intervention effects (e.g., individual, group, or community)	✓	6–7; Supp. 39–43
		• If the unit of analysis differs from the unit of assignment, the analytical method used to account for this (e.g., adjusting the standard error estimates by the design effect or using multilevel analysis)	N/A	
Statistical methods	11	• Statistical methods used to compare study groups for primary methods outcome(s), including complex methods for correlated data	✓	6-7; Supp. 39-43
		• Statistical methods used for additional analyses, such as subgroup analyses and adjusted analysis	✓	7; Supp. 40-43
		• Methods for imputing missing data, if used	✓	7; Supp. 43 ****

		• Statistical software or programs used	✓	6; Supp. 39
**RESULTS**				
Participant flow	12	• Flow of participants through each stage of the study: enrollment, assignment, allocation and intervention exposure, follow-up, analysis (a diagram is strongly recommended)	✓	8-9, Fig. 1
		o Enrollment: the numbers of participants screened for eligibility, found to be eligible or not eligible, declined to be enrolled, and enrolled in the study	✓	8-9, Fig. 1
		o Assignment: the numbers of participants assigned to a study condition	✓	8-9, Fig. 1
		o Allocation and intervention exposure: the number of participants assigned to each study condition and the number of participants who received each intervention	✓	8-9, Fig. 1
		o Follow-up: the number of participants who completed the follow-up or did not complete the follow-up (i.e., lost to follow-up), by study condition	✓	8-9, Fig. 1
		o Analysis: the number of participants included in or excluded from the main analysis, by study condition	✓	8-9, 14-18, Fig. 1
		• Description of protocol deviations from study as planned, along with reasons	✓	6,26 *****
Recruitment	13	• Dates defining the periods of recruitment and follow-up	✓	3, 8; Supp. 33
Baseline data	14	• Baseline demographic and clinical characteristics of participants in each study condition	✓	9-14; Tables 1–2
		• Baseline characteristics for each study condition relevant to specific disease prevention research	✓	9-14; Tables 1–2
		• Baseline comparisons of those lost to follow-up and those retained, overall and by study condition	✓	Supp. Table S4******, p.54
		• Comparison between study population at baseline and target population of interest	N/A	
Baseline equivalence	15	• Data on study group equivalence at baseline and statistical methods used to control for baseline differences	✓	10-14;7; ; Supp. 41-3
Numbers analyzed	16	• Number of participants (denominator) included in each analysis for each study condition, particularly when the denominators change for different outcomes; statement of the results in absolute numbers when feasible	✓	14-26; Tables 3–10
		• Indication of whether the analysis strategy was "intention to treat" or, if not, description of how non-compliers were treated in the analyses	✓	6

Outcomes and estimation	17	• For each primary and secondary outcome, a summary of results for each estimation study condition, and the estimated effect size and a confidence interval to indicate the precision	✓	14-25; Tables 3–10
		• Inclusion of null and negative findings	✓	14-26
		• Inclusion of results from testing pre-specified causal pathways through which the intervention was intended to operate, if any	N/A*******	
Ancillary analyses	18	• Summary of other analyses performed, including subgroup or restricted analyses, indicating which are pre-specified or exploratory	✓	15-25; Supp. 40-43, 54-64
Adverse events	19	• Summary of all important adverse events or unintended effects in each study condition (including summary measures, effect size estimates, and confidence intervals)	✓	26
**DISCUSSION**				
Interpretation	20	• Interpretation of the results, taking into account study hypotheses, sources of potential bias, imprecision of measures, multiplicative analyses, and other limitations or weaknesses of the study	✓	26-30
		• Discussion of results taking into account the mechanism by which the intervention was intended to work (causal pathways) or alternative mechanisms or explanations	✓	26-30
		• Discussion of the success of and barriers to implementing the intervention, fidelity of implementation	✓	6, 26
		• Discussion of research, programmatic, or policy implications	✓	28-31
Generalizability	21	• Generalizability (external validity) of the trial findings, taking into account the study population, the characteristics of the intervention, length of follow-up, incentives, compliance rates, specific sites/settings involved in the study, and other contextual issues	✓	29-31
Overall evidence	22	• General interpretation of the results in the context of current evidence and current theory	✓	26-31

*Not applicable because this TREND item refers specifically to theories used in designing behavioral interventions; the present study was based on a biological mechanistic rationale rather than a behavioral theory.

**Not applicable because no psychometric or other validated measurement instruments were used; study outcomes were based on standardized clinical and laboratory assessments.

***The study was non-randomized and open-label; participants and treating clinicians were not blinded to study-group allocation.

****Missing-data handling and sensitivity analyses, including multiple imputation, are described in the Supplementary Methods.

*****No major protocol deviations affecting intervention exposure were observed; adherence and treatment discontinuation are reported in the Methods and Results.

******Characteristics of recipients with and without available Month 3 UACR are presented in Supplementary Table S4.

*******Not applicable because mechanistic mediators were not directly measured; the proposed biological pathway was therefore not formally tested.
